# Biomimetic Targeted Co‐Delivery System Engineered from Genomic Insights for Precision Treatment of Osteosarcoma

**DOI:** 10.1002/advs.202410427

**Published:** 2024-11-18

**Authors:** Tianqi Luo, Zhijin Fan, Anyu Zeng, Anqi Wang, Yuanwei Pan, Yanyang Xu, Hongmin Chen, Weiqing Chen, Dingmeng Nie, Jiaming Lin, Anfei Huang, Ming Gong, Yufeng Huang, Yun Ding, Xiaojun Zhu, Lang Rao, Jin Wang

**Affiliations:** ^1^ Department of Musculoskeletal Oncology State Key Laboratory of Oncology in South China, Guangdong Provincial Clinical Research Center for Cancer Sun Yat‐sen University Cancer Center Guangzhou 510060 China; ^2^ Institute for Engineering Medicine Kunming Medical University Kunming 650500 China; ^3^ Institute of Chemical Biology Shenzhen Bay Laboratory Shenzhen 518132 China; ^4^ Department of Joint Surgery Guangzhou First People's Hospital School of Medicine South China University of Technology Guangzhou 510060 China; ^5^ Department of Pediatric Orthopaedics Guangzhou Women and Children's Medical Center Guangdong Provincial Clinical Research Center for Child Health Guangzhou Medical University Guangzhou 510630 China; ^6^ Department of Breast Oncology State Key Laboratory of Oncology in South China, Guangdong Provincial Clinical Research Center for Cancer Sun Yat‐sen University Cancer Center Guangzhou 510060 China

**Keywords:** B7H3, bioinformatics, Homologous recombination repair, nanoparticles, osteosarcoma

## Abstract

The high heterogeneity and severe side effects of chemotherapy are major factors contributing to the failure of osteosarcoma treatment. Herein, a comprehensive genomic analysis is conducted, and identified two prominent characteristics of osteosarcoma: significant cyclin‐dependent kinases 4 (CDK4) amplification and homologous recombination repair deficiency. Based on these findings, a co‐delivery system loaded with CDK4/6 inhibitors and poly ADP‐ribose polymerase (PARP) inhibitors is designed. By employing metal–organic frameworks (MOFs) as carriers, issue of drug insolubility is effectively addressed, while also enabling controlled release in response to the tumor microenvironment. To enhance targeting capability and biocompatibility, the MOFs are further coated with a bio‐membrane targeting B7H3. This targeted biomimetic co‐delivery system possesses several key features: 1) it can precisely target osteosarcoma with high B7H3 expression; 2) the combination of CDK4/6 inhibitors and PARP inhibitors exhibits synergistic effects, significantly impairing tumor's DNA repair capacity; and 3) the system has the potential for combination with photodynamic therapy, amplifying DNA repair defects to maximize tumor cell eradication. Furthermore, it is observed that this co‐delivery system can activate immune microenvironment, increasing CD8^+^ T cell infiltration and converting osteosarcoma from an immune‐cold to an immune‐hot tumor. In summary, the co‐delivery system is an effective therapeutic strategy and holds promise as a novel approach for osteosarcoma treatment.

## Graphical abstract

Based on comprehensive genomic analysis, a targeting B7H3 co‐delivery system loaded with cyclin‐dependent kinases 4/6 (CDK4/6) inhibitors and poly ADP‐ribose polymerase (PARP) inhibitors is developed. Through combined photodynamic therapy, the co‐delivery system can eliminate tumor cells through dual synergistic mechanisms while activating immune microenvironment via the cGAS‐STING pathway.

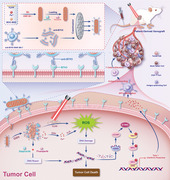

## 1 Introduction

Osteosarcoma is the most common primary malignant bone tumor, primarily affecting children and adolescents.^[^ ^1^ ^]^ The combination of chemotherapy and surgery is the current standard treatment for osteosarcoma in clinical practice. However, due to the substantial heterogeneity of osteosarcoma, many patients still experience metastasis and recurrence as a result of developing drug resistance.^[^ ^2^ ^]^ Moreover, chemotherapy is frequently associated with severe side effects, as drugs like cisplatin and doxorubicin not only target cancer cells but also damage normal cells, which results in significant adverse effects such as myelosuppression, cardiotoxicity, and nephrotoxicity.^[^ ^3^ ^]^ The presence of these side effects often necessitates dose limitations, which can lead to suboptimal therapeutic outcomes. Consequently, the therapeutic outcomes for osteosarcoma have not significantly improved over the past four decades, underscoring the pressing need for novel and effective therapeutic approaches.

Nowadays, the application of bioinformatics in cancer treatment has become increasingly pivotal. By analyzing patients' genomic information, key mutations driving tumor growth can be identified, allowing for the selection of individualized, targeted therapies.^[^ ^4^ ^]^ Moreover, single‐cell RNA‐seq enables the exploration of gene expression differences among various cell populations within the tumor, providing deeper insights into tumor heterogeneity and its response to treatment.^[^ ^5^ ^]^ At the initial stage of the study, genomic analysis of osteosarcoma identified cyclin‐dependent kinases 4/6 (CDK4/6) inhibitor and poly ADP‐ribose polymerase (PARP) inhibitor as potential therapeutic agents. This combination has been demonstrated to synergistically inhibit multiple DNA repair pathways in osteosarcoma. Concurrently, our center has initiated a Phase II clinical trial to investigate the combination therapy of Dalpiciclib (CDK4/6 inhibitor)^[^ ^6^ ^]^ and Fluzoparib (PARP inhibitor)^[^ ^7^ ^]^ for advanced metastatic bone and soft tissue sarcoma patients (NCT05952128). However, during follow‐up, it was observed that a subset of patients did not benefit from this therapeutic strategy, and combined therapy resulted in significant adverse effects due to the long‐term course (21 consecutive days).

In recent years, nanomaterials have demonstrated notable advantages as drug delivery vehicles, particularly by improving drug solubility, minimizing peripheral side effects, and thereby improving therapeutic efficacy in vivo.^[^ ^8^, ^9^, ^10^ ^]^ MOFs, in particular, stand out due to their high porosity and large surface area, which facilitate substantial drug loading.^[^ ^11^, ^12^, ^13^ ^]^ On the one hand, metal–organic frameworks (MOFs) allow controlled drug release in precise ratios and at specific conditions, which is especially beneficial for combination therapies and can maximize synergistic effects. On the other hand, Meso‐tetra (4‐carboxyphenyl) porphine (TCPP)‐based MOFs generate reactive oxygen species (ROS) under laser irradiation, which induces DNA damage in tumor cells and synergizes with combined therapy to effectively kill cancer cells.^[^ ^12^ ^]^ Consequently, MOFs can significantly potentiate the therapeutic efficacy of Dalpiciclib and Fluzoparib in osteosarcoma through effective drug loading and enhanced synergy with photodynamic therapy (PDT).

However, the mineral‐rich dense bone matrix can pose significant barriers to the penetration and accumulation of drugs within osteosarcomas. Therefore, enhancing the active targeting capability of MOFs becomes imperative. Cell membrane‐derived nanovesicles offer distinct advantages in tumor‐targeted therapy.^[^ ^14^, ^15^, ^16^ ^]^ First, they possess inherent biocompatibility, enabling immune evasion, thereby prolonging their circulation time within the body. Second, genetic engineering approaches enable the expression of specific receptors on these nanovesicles, facilitating active targeting of nanoparticles.^[^ ^17^ ^]^ Bioinformatics analysis identified elevated B7H3 expression in osteosarcoma compared to normal tissues, suggesting its potential as a therapeutic target for osteosarcoma. Therefore, by coating MOFs with cell membranes expressing anti‐B7H3, the targeting capability toward osteosarcoma can be specifically enhanced, thereby improving the efficacy of the combined therapy.

Herein, we pioneered the integration of bioinformatics and nanomedicine to develop a TCPP ‐MOFs loaded with Dalpiciclib and Fluzoparib. Through coating with genetically engineered macrophage membranes, a targeted biomimetic co‐delivery system (TBCDS) was ultimately constructed. This co‐delivery system not only enables precision treatment tailored to the genomic characteristics and vulnerabilities of osteosarcoma but also exhibits specific targeting capabilities. Notably, it has the potential to effectively eliminate tumor cells through synergy with PDT. Overall, this novel co‐delivery system represents a promising therapeutic approach for osteosarcoma.

## 2 Results and Discussion

### 2.1 Design and Validation of Targeting B7H3 in Osteosarcoma

**Figure 1:**
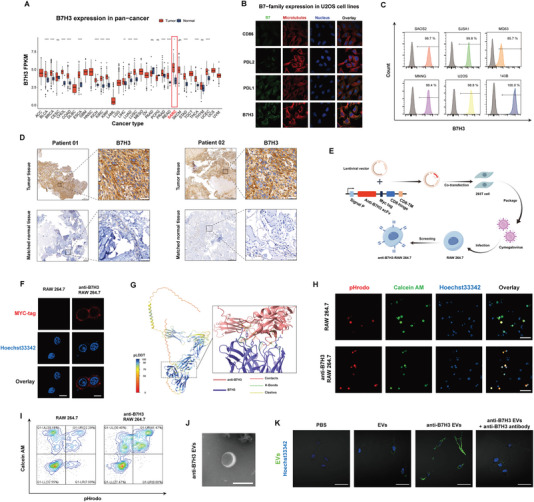
Bioinformatics Analysis of B7H3 Expression and Functional Validation of Anti‐B7H3 in Osteosarcoma. A) B7H3 expression in Pan‐cancer in TCGA database. B) Immunofluorescence of B7‐family expression on U2OS cells in HPA database. C) Flow Cytometry Analysis of B7H3 in types of Osteosarcoma cell lines. D) Immunohistochemistry of B7H3 in osteosarcoma patient and matched adjacent normal tissues from Sun Yat‐sen University Cancer Center (Scale bar = 50 µm). E) Schematic representation of the lentiviral transduction process used to generate RAW264.7 cells expressing the anti‐B7H3 protein. F) Confocal laser scanning microscope (CLSM) of MYC‐Tag in transduced RAW264.7 cells (Scale bar = 10 µm). G) Structure prediction of the anti‐B7H3 antibody binding to B7H3 using AlphaFold 3. H) Representative CLSM images of phagocytosis assay for anti‐B7H3 expressing RAW264.7 cells and K7M2 cells (n = 3; Scale bar = 50 µm). I) Flow cytometry result of phagocytosis assay for anti‐B7H3 expressing RAW264.7 cells and K7M2 cells (n = 3). J) Electron Microscopy of Extracellular vesicles (Scale bar = 100 nm). K) Representative CLSM images of DIO‐labeled anti‐B7H3 EVs and SJSA1 cells (n = 3; Scale bar = 50 µm). Results are all presented as the mean ± SD. The statistical analysis was performed using a one‐way ANOVA, *p < 0.05, **p < 0.01, ***p < 0.001.

Building on previous research findings, we focused on B7H3 (CD276), one of the most highly expressed molecules on the osteosarcoma cell membrane.^[^ ^18^ ^]^ B7H3, a member of the B7 family, is widely expressed in various cancers. Transcriptome analysis of thirty‐three tumor types from the Cancer Genome Atlas (TCGA) database^[^ ^19^ ^]^ revealed that B7H3 expression was elevated in most tumors compared to normal tissues (**Figure** **1**A). Notably, the TCGA‐SARC cohort exhibited the highest levels of B7H3 expression, suggesting its potential as a therapeutic target for sarcomas. Further investigation using the Human Protein Atlas (HPA), a comprehensive proteomics database,^[^ ^20^ ^]^ confirmed that B7H3 is highly expressed in the osteosarcoma cell line U2OS. The expression of B7H3 was found to be significantly higher than that of other B7 family members, such as PDL1 and PDL2, and was primarily localized on the cell membrane (Figure 1B). Flow cytometry analysis of six osteosarcoma cell lines revealed that B7H3 is localized on the cell membrane, with a positivity rate exceeding 90% (Figure 1C). To further explore the therapeutic potential of B7H3, its expression levels were systematically compared in tumor tissues and matched adjacent normal tissues from osteosarcoma patients at our institution. The results demonstrated high B7H3 expression in tumor tissues, while expression was low or undetectable in normal tissues (Figure 1D).

To develop biomimetic nanoparticles (BNPs) with targeting capabilities, a sequence encoding the anti‐B7H3 gene was designed, inspired by chimeric antigen receptor (CAR) T cells.^[^ ^21^, ^22^ ^]^ This sequence included the anti‐B7H3 and MYC‐tag on the extracellular side of the cell membrane, anchored by a hinge and transmembrane region (TMR). Lentiviral transduction was used to express the target protein in RAW264.7 cells, a widely utilized mouse macrophage cell line for the preparation of biomembrane materials^[^ ^21^, ^23^, ^24^ ^]^ (Figure 1E). Then, the presence of the antibody was validated through indirect detection of protein tags (MYC‐tag). Immunofluorescence confirmed the successful expression of anti‐B7H3 on the cell membrane of RAW264.7 (Figure 1F). Flow cytometry and western blot analysis further validated the expression of anti‐B7H3, showing a distinct protein band at the expected molecular weight (Figure S1, Supporting Information). To predict the structure of the anti‐B7H3, AlphaFold 3, an advanced AI‐driven model for protein structure prediction,^[^ ^25^ ^]^ was employed. Compared to other models, AlphaFold 3 exhibited superior accuracy and confidence in its predictions. The results showed that the predicted molecular structures exhibited high confidence, with complementary binding sites identified between the two protein molecules. Using molecular docking analysis, the interactions, clashes, and hydrogen bonds between anti‐B7H3 and B7H3 were visualized, revealing a strong interaction that supports the potential efficacy of the biomimetic material for targeted therapy (Figure 1G).

To assess the function of anti‐B7H3, a phagocytosis assay was conducted using RAW264.7 macrophages expressing anti‐B7H3.^[^ ^26^ ^]^ In Figure 1H, Calcein AM‐labeled RAW cells were co‐cultured with pHrodo‐labeled K7M2 cells. The pHrodo, a pH‐sensitive dye that fluoresces in acidic conditions such as within intracellular setting, served as a reliable marker for phagocytosis. Cells that are double‐positive for Calcein AM and pHrodo represent macrophages that have phagocytosed tumor cells. Immunofluorescence results showed that targeting‐B7H3 RAW cells exhibited a significantly higher proportion of double‐positive cells compared to controls. Additionally, flow cytometry analysis revealed that RAW cells expressing anti‐B7H3 showed a higher phagocytosis percentage after co‐incubation with tumor cells compared to the control group (53.07 ± 2.18% vs 37.38 ± 0.72%), demonstrating effective recognition and binding of target cells, thereby enhancing phagocytic capacity (Figure 1I; Figure S2, Supporting Information). Additionally, extracellular vesicles (EVs)^[^ ^16^, ^17^ ^]^ derived from RAW cells were prepared (Figure 1J) and labeled with DIO dye. Upon incubation with SJSA1 cells, anti‐B7H3 EVs accumulated around the tumor cell membrane more significantly than control EVs. This accumulation was reversed by pretreatment with an anti‐B7H3 neutralizing antibody, indicating specific binding mediated by anti‐B7H3 (Figure 1K). Previous studies have also highlighted the efficacy of targeting B7H3 in tumor treatments. Previous studies have demonstrated that B7H3‐targeted therapies, such as Antibody‐Drug Conjugate (ADC) drugs and CAR‐T therapy, have shown some efficacy in the treatment of bone and soft tissue sarcoma.^[^ ^27^, ^28^, ^29^ ^]^ Combined with our findings, this suggests that B7H3 is a promising therapeutic target for osteosarcoma.

### 2.2 Genomic Insights into CDK4 and Its Therapeutic Potential in Osteosarcoma

**Figure 2:**
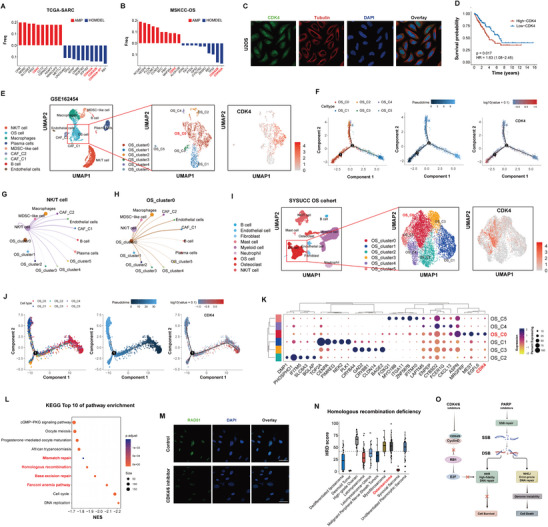
Genomic Characteristics and Therapeutic Strategies of Osteosarcoma. A) Top ten genes with the highest amplification and deletion rates in the TCGA‐SARC cohort (n = 255). B) Top ten genes with the highest amplification and deletion rates in the MSKCC cohort (n = 129). C) Expression of CDK4 in U2OS cell lines from the HPA database. D) Survival analysis of CDK4 in TCGA‐SARC cohort (n = 248). E) Dimensionality reduction analysis of single‐cell sequencing data from the GSE162454 dataset (n = 6). F) Pseudotime analysis of different tumor cell clusters from the GSE162454 dataset. G) Cell chat analysis between NK/T cells and other cell populations in the GSE162454 dataset. H) Cell chat analysis between OS_cluster0 and other cell populations in the GSE162454 dataset. I) Dimensionality reduction analysis of single‐cell sequencing data from the SYSUCC OS cohort (n = 8). J) Pseudotime analysis of different tumor cell clusters from the SYSUCC OS cohort. K) Bubble plot of highly expressed genes across different cell subpopulations in SYSUCC OS cohort. L) GSEA analysis of SJSA1 cell line treated with CDK4/6 inhibitors (n = 3). M) Representative CLSM images of RAD51 levels in SJSA1 cell line treated with CDK4/6 inhibitors (n = 3; Scale bar = 50 µm). N) Quantitation analysis of HRD score in the TCGA‐OV, TCGA‐SARC and TARGET‐OS cohorts (n = 377). O) Representation of the synergistic mechanisms of CDK4/6 inhibitors and PARP inhibitors.

To achieve the most precise therapeutic effect, gene mutations in the TCGA‐SARC cohort were analyzed (**Figure** **2**A). Among the top ten genes with the highest amplification rates, CDK4 was identified as a key focus. CDK4, a cyclin‐dependent kinase, forms a complex with CDK6 and Cyclin D to drive the G1 to S phase transition of the cell cycle, with its overactivation being associated with tumor development and progression.^[^ ^30^ ^]^ Additionally, CDKN2A and CDKN2B, identified among the top ten genes with the highest deletion rates, act as tumor suppressor genes inhibiting the CDK4‐Cyclin D complex, thus playing a crucial role in cell cycle regulation.^[^ ^31^ ^]^

Similar findings were observed in the Memorial Sloan Kettering Cancer Center (MSKCC) osteosarcoma cohort^[^ ^32^ ^]^ (Figure 2B), suggesting that cell cycle dysregulation is a prominent genomic characteristic of osteosarcoma. Further analysis from the HPA database revealed significant CDK4 expression in osteosarcoma cell lines (Figure 2C). Survival analysis of TCGA‐SARC indicated that high CDK4 expression was correlated with worse overall survival in sarcoma patients (p value = 0.017; Figure 2D).

Due to the high heterogeneity of osteosarcoma, tumor tissue sequencing may not fully capture the true landscape. Therefore, single‐cell sequencing data of osteosarcoma (GSE162454) from the GEO database was analyzed.^[^ ^33^ ^]^ Dimensionality reduction analysis identified nine cell populations, including osteosarcoma cells, macrophages, NK/T cells, fibroblasts, and others (Figure 2E). Further clustering of osteosarcoma cells (OS cell) revealed six subpopulations, among which the largest cluster (cluster 0) exhibited high CDK4 expression (Figure 2E). Pseudotime analysis indicated that the high CDK4‐expressing cluster 0 was positioned at the beginning of the cell differentiation trajectory, indicating potential tumor stemness (Figure 2F). Moreover, CellChat analysis revealed diminished signaling interactions between NK/T cells and Cluster 0 cells, while Cluster 0 cells exhibited enhanced communication with cancer‐associated fibroblasts (CAFs). CAFs are known to modulate the composition and structure of the extracellular matrix (ECM), thereby increasing the density of tumor tissue and creating a physical barrier that hinders the penetration and distribution of chemotherapeutic drugs^[^ ^34^ ^]^ (Figure 2G,H). To further investigate the association between CDK4 and stem‐like cells in osteosarcoma, single‐cell data from eight untreated osteosarcoma patients of our center were analyzed.^[^ ^35^ ^]^ Dimensionality reduction analysis identified six distinct tumor cell clusters (Figure 2I). Consistent with previous datasets, pseudotime analysis indicated that Cluster 0, located at the initial stage of cell differentiation, exhibited high expression of CDK4 (Figure 2J,K). These findings showed that high amplification of CDK4 is closely related to the occurrence and development of osteosarcoma, indicating that CDK4/6 inhibitors might be effective for osteosarcoma.^[^ ^36^ ^]^ However, in clinical practice, resistance to CDK4/6 inhibitors frequently develops through various mechanisms.^[^ ^37^ ^]^ As a result, current therapeutic approaches primarily focus on combination regimens based on CDK4/6 inhibitors.^[^ ^38^, ^39^, ^40^, ^41^, ^42^, ^43^ ^]^

### 2.3 Exploration of Combination Therapies: CDK4/6 and PARP Inhibitors for Osteosarcoma

To identify candidates for combination therapy with CDK4/6 inhibitors, transcriptomic sequencing and Gene set enrichment analysis (GSEA) were performed on the osteosarcoma cell line SJSA1 treated with CDK4/6 inhibitors.^[^ ^44^ ^]^ In addition to the observed downregulation of DNA replication and cell cycle pathways, pathways related to homologous recombination repair also exhibited significant downregulation (Figure 2L). In the next, immunofluorescence assessment of RAD51, a core homologous recombination repair (HRR) gene,^[^ ^45^ ^]^ revealed a notable decrease of RAD51 levels in CDK4/6 inhibitor‐treated osteosarcoma cells (Figure 2M), suggesting impaired HRR capability. The decline in HRR capability leads to the accumulation of DNA damage and chromosomal aberrations, thereby contributing to genomic instability. However, certain tumors, such as ovarian cancer, benefit from PARP inhibitors due to their high homologous recombination deficiency (HRD) characteristics.^[^ ^46^ ^]^ Therefore, we hypothesized that CDK4/6 inhibition could further exacerbate this DNA repair defect, thereby enhancing the sensitivity of high HRD tumors to PARP inhibitors. Accordingly, an analysis was performed on genomic instability features, including loss of heterozygosity (LOH), telomeric allelic imbalance (TAI), large‐scale state transitions (LST), and HRD scores in the TCGA‐SARC and TARGET‐OS cohorts, with the ovarian cancer cohort serving as a positive control. Consistent with previous studies,^[^ ^47^ ^]^ sarcoma patients, particularly those with osteosarcoma, exhibited high levels of LOH, TAI, LST, and HRD scores (Figure S3, Supporting Information, Figure 2N). Moreover, survival analysis of the TCGA‐SARC cohort demonstrated a significant correlation between high HRD scores and poor prognosis (p value = 0.047; Figure S4, Supporting Information). These results suggested that osteosarcoma may be sensitive to PARP inhibitors, with CDK4/6 inhibitors potentially enhancing this sensitivity.^[^ ^48^ ^]^ In contrast, osteosarcomas with RB1 deficiency exhibit heightened sensitivity to PARP inhibitors,^[^ ^49^ ^]^ despite RB1 loss being a major mechanism of resistance to CDK4/6 inhibitors.^[^ ^37^ ^]^ This complementary effect also supports the combined use of PARP inhibitors and CDK4/6 inhibitors for the treatment of osteosarcoma (Figure 2O). Importantly, the efficacy of the combination of these two drugs has been demonstrated across various types of tumors.^[^ ^48^, ^50^, ^51^, ^52^, ^53^, ^54^ ^]^

### 2.4 Development and Characterization of Nanoparticles for Enhanced Drug Delivery

**Figure 3:**
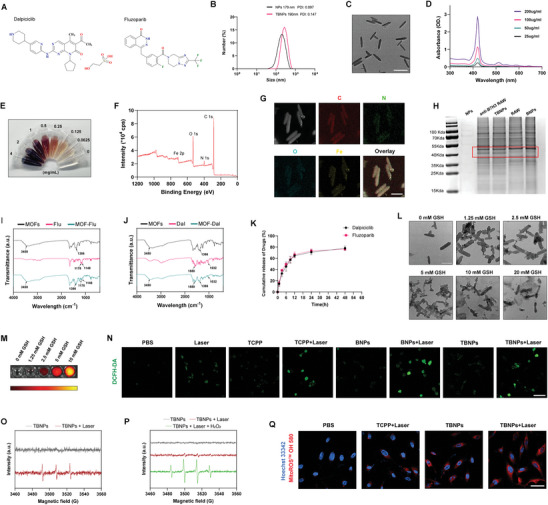
Preparation and Characterization of Nanoparticles. A) Chemical structures of Dalpiciclib and Fluzoparib. B) Particle size distribution of the nanoparticles. C) TEM images of the nanoparticles (Scale bar = 200 nm). D) UV–vis absorption spectra of the nanoparticles. E) Visual representation of nanoparticle solutions at different concentrations. F) The XPS spectrum of nanoparticles. G) EDS spectra of C, N, O, and Fe elements within the nanoparticles (Scale bar = 100 nm). H) Coomassie Brilliant Blue staining of protein expression profiles for NPs (nanoparticles; MOFs), BNPs (Biomimetic nanoparticles), and TBNPs (Targeted biomimetic nanoparticles). I) and (J) FT‐IR analysis of MOFs, Dalpiciclib and Fluzoparib. K) Drug release efficiency under incubation with GSH over time (n = 3). L) Representative TEM images of NPs morphology at varying GSH concentrations (Scale bar = 200 nm). M) Fluorescence intensity of NPs at varying GSH concentrations. N) Representative CLSM images of ROS levels under different treatment (n = 3; Scale bar = 50 µm). O) ESR spectra with TEMP probes of TBNPs with or without irradiation with laser. P) ESR spectra with DMPO probes of TBNPs with or without the addition of H2O2 or irradiation with laser. Q) Representative confocal microscopy images of hydroxyl radicals detection in SJSA1 cells treated with PBS, TCPP + Laser, TBNPs, and TBNPs + Laser (Scale bar = 50 µm). Results are all presented as the mean ± SD. The statistical analysis was performed using a one‐way ANOVA, *p < 0.05, **p < 0.01, ***p < 0.001.

Based on the above results from the osteosarcoma genome analysis, Dalpiciclib and Fluzoparib were selected as candidates for osteosarcoma treatment. Clinical studies have shown that both CDK4/6 inhibitors and PARP inhibitors benefit patients with advanced bone and soft tissue sarcoma when used individually.^[^ ^55^, ^56^ ^]^ Similarly, in preclinical models of bone and soft tissue sarcoma, we observed a synergistic effect between the two inhibitors (Figure S5, Supporting Information). Consequently, clinical trials were initiated to further investigate this combination therapy. However, during follow‐up, it was observed that a subset of patients failed to achieve clinical benefit from the treatment and experienced severe toxicities. In consideration of the lipophilic nature of these two drugs (**Figure** **3**A), their absorption in the body is somewhat limited, which in turn restricts their efficacy.^[^ ^57^ ^]^ To address this, MOFs were employed as carriers to enhance drug solubility and enable precise drug release. To construct ideal MOFs, TCPP and FeCl_3_ were used to prepare GSH‐responsive Fe‐MOFs. As shown in Figure 3B, the synthesized nanoparticles (NPs) had a particle size of 179 nm and a polydispersity index (PDI) of 0.097. After coating with a cell membrane, the particle size increased to 190 nm with a PDI of 0.147. In addition, the particle size in various solvent systems remained stable over seven days, indicating that the synthesized material has high stability (Figure S6, Supporting Information). In Figure 3C, transmission electron microscopy (TEM) showed that the NPs length matched our measurements, and their rod‐shaped morphology was advantageous for penetrating tumor tissue.^[^ ^58^, ^59^ ^]^ Furthermore, the UV‐vis absorption spectrum of the nanoparticles exhibited two prominent peaks: a strong Soret band in the range of 400–500 nm and a weak Q band in the range of 500–700 nm (Figure 3D). In Figure 3E, the appearance of nanoparticles in aqueous solution at different concentrations showed a brownish‐red color similar to that of TCPP. To investigate the coordination between metal ions and organic ligands, X‐ray Photoelectron Spectroscopy (XPS) analysis was performed on NPs (Fe‐MOFs). The results identified the presence of four elements (C, N, O, and Fe) in Fe‐MOFs (Figure 3F). In the C 1s spectrum, peaks at 284.8, 286.5, and 289.0 eV correspond to C─C/C─H, C─O, and O─C═O bonds (Figure S7A, Supporting Information), indicating single and double bonds between carbon, hydrogen, and oxygen. The O 1s spectrum exhibited C═O and C─OH peaks at 531.9 and 533.7 eV (Figure S7B, Supporting Information). In the N 1s spectrum, peaks at 397.7 and 399.9 eV correspond to C═NH─C and C─NH─C environments, respectively. The C─NH─C peak represents single‐bonded nitrogen atoms connected to two carbon atoms within the porphyrin ring, while the C═NH─C peak indicates a nitrogen atom double‐bonded to one carbon and single‐bonded to another carbon and a hydrogen atom, forming a coordination bond (Figure S7C, Supporting Information). Importantly, the Fe 2p spectrum was fitted into three pairs of symmetric peaks, where binding energies at 711.4 and 724.7 eV correspond to Fe^2^⁺ 2p3/2 and Fe^2^⁺ 2p1/2, while the peaks at 713.4 and 725.9 eV are assigned to Fe^3^⁺ 2p3/2 and Fe^3^⁺ 2p1/2 (Figure S7D, Supporting Information). Additionally, satellite peaks at 717.5 and 731.1 eV, typically associated with Fe 2p, are observed at higher binding energies due to internal electronic transitions within the iron atoms. The iron element primarily exists in both divalent and trivalent states, with peak broadening or multiple peaks resulting from the formation of coordination bonds, which alter the distribution of electronic energy states due to changes in the coordination environment. These results confirm that the iron ions form coordination bonds with the carbonyl groups of TCPP, further supporting the formation and chemical stability of Fe‐TCPP.

Then, energy dispersive spectrometer (EDS) analysis further confirmed the presence of C, N, O, and Fe in Fe‐MOFs (Figure 3G). To endow materials with the ability to target osteosarcoma, the anti‐B7H3 macrophage membrane was used to coat the NPs to develop targeted biomimetic nanoparticles (TBNPs). Coomassie Brilliant Blue staining showed that the protein profiles of nanoparticles (NPs/MOFs), biomimetic nanoparticles (coated with blank macrophage membrane; BNPs), and TBNPs were consistent with their source membrane, with TBNPs exhibiting significant anti‐B7H3 expression (Figure 3H). In the Figure S8 (Supporting Information), the ZETA potential levels of NPs, BNPs, and TBNPs were measured at 31.3 ± 2.52 mV, −25.3 ± 1.53 mV, and −25 ± 2.65 mV, respectively, indicating that the suspension system of NPs exhibits remarkablestability, with the particles being less prone to aggregation or sedimentation.

To confirm the successful loading of both drugs into the NPs, Fourier transform infrared spectrum (FT‐IR) analysis was performed. As shown in Figures 3I,J, the NPs loaded with either Fluzoparib or Dalpiciclib exhibited characteristic peaks similar to those of the respective drugs, indicating successful drug incorporation into the NPs. High‐performance liquid chromatography (HPLC) was utilized to quantify the encapsulation efficiency and drug loading capacity of the nanomaterial for two drugs: Dalpiciclib and Fluzoparib. Due to the overlapping absorption spectra of the two drugs, it was challenging to distinguish them at a single wavelength. Therefore, separate analyses were conducted at different wavelengths to accurately measure each drug. Dalpiciclib was detected at a wavelength of 360 nm, while Fluzoparib was analyzed at 250 nm (Figure S9, Supporting Information). This method ensured precise quantification of each drug's concentration within the nanomaterial, enabling accurate evaluation of the co‐delivery system's performance.^[^ ^60^, ^61^ ^]^ HPLC results showed that the solutions of nanoparticles loaded with Dalpiciclib or Fluzoparib had peaks overlapping with those of the respective drugs, indicating successful drug loading. The encapsulation efficiency was 75.23% for Dalpiciclib and 79.01% for Fluzoparib (Table S1, Supporting Information). In Figure 3K, HPLC evaluation of drug release efficiency showed that both drugs achieved optimal release levels within 24 h under incubation with 10 mM GSH.

### 2.5 Enhanced ROS Generation in Tumors via Nanoparticles’ Dual Mechanism

The tumor microenvironment is typically characterized by elevated levels of GSH and hydrogen peroxide (H₂O₂). Glutathione aids tumor cells in resisting oxidative stress and drug toxicity, while hydrogen peroxide, a byproduct of the high metabolic activity of tumor cells, plays a role in promoting tumor growth and invasion.^[^ ^62^ ^]^ As illustrated in Figure 3L, TEM images of the NPs incubated with varying concentrations of GSH demonstrated a significant loss of original morphology at a concentration of 10 mM, indicating GSH‐responsiveness. TCPP is a photosensitizer known for its ability to generate ROS under light irradiation. To further evaluate the GSH‐responsive release of TCPP from NPs, In Vivo Imaging was employed to measure TCPP fluorescence. This experiment utilized the principle of Aggregation‐Caused Quenching (ACQ),^[^ ^63^ ^]^ where the fluorescence of TCPP is quenched when aggregated within intact NPs. Upon NPs degradation, TCPP is released, resulting in fluorescence emission. The results showed a positive correlation between TCPP fluorescence intensity and increasing GSH concentration, confirming the material's capacity for GSH‐triggered TCPP release. (Figure 3M).

Then, the ability of the NPs to generate ROS under irradiation was investigated using the DCFH‐DA probe. Under 660 nm laser irradiation, the fluorescence intensity of DCFH‐DA increased with rising GSH concentrations in NPs, indicating that more TCPP was being released, leading to higher ROS production (Figure S10, Supporting Information). Although GSH can consume ROS, the presence of iron ions simultaneously depleted GSH, thereby weakening this effect. Furthermore, the ability of NPs to induce intracellular ROS levels was further investigated in tumor cells. Interestingly, the results revealed that NPs (BNPs and TBNPs) exposed to laser irradiation produced higher levels of ROS compared to the TCPP + laser group (Figure 3N). We hypothesize that this effect may be attributed to the Fenton reaction occurring within the tumor cells due to the presence of iron ions in the NPs, leading to the generation of additional hydroxyl radicals.^[^ ^64^, ^65^ ^]^

Hydroxyl radicals (•OH), as the most reactive type of ROS, can efficiently damage tumor cell DNA, membrane lipids, and proteins, causing irreversible oxidative damage and inducing apoptosis or necrosis. Compared to other ROS types (such as singlet oxygen and superoxide), •OH exhibits stronger reactivity and faster action, making them more effective in disrupting tumor cell structures. This high reactivity is particularly advantageous in chemodynamic therapy (CDT), where •OH demonstrates significant anti‐tumor effects.^[^ ^66^ ^]^ To verify this hypothesis, the level of singlet oxygen (^1^O_2_) and •OH induced by TBNPs was first assessed using electron spin resonance spectroscopy (ESR). As shown in Figure 3O, the ESR spectrum displays characteristic ^1^O_2_ signals following exposure to laser, indicating the ability of TBNPs to generate ^1^O_2_ under irradiation. Additionally, the SOSG probe was utilized to assess the generation of ^1^O_2_ across different groups. The results indicated that, in the absence of irradiation, all three groups exhibited limited capability to produce ^1^O_2_. However, upon the introduction of laser, the fluorescence intensity in the TCPP group significantly increased. However, the TBNPs also generated a substantial amount of ^1^O_2_ under laser irradiation after GSH pre‐treatment (Figure S11, Supporting Information). In the ESR spectrum shown in Figure 3P, laser irradiation resulted in the generation of trace amounts of •OH by the TBNPs. However, the addition of H_2_O_2_ significantly amplified the signal of the generated •OH, indicating that a Fenton reaction occurred within this system. Furthermore, colorimetric assay of •OH using 3, 3′, 5, 5′‐tetramethyl‐benzidine (TMB) further corroborated this finding (Figure S12, Supporting Information). Finally, MitoROS OH580 was utilized to assess the levels of •OH generated by Fenton reaction in tumor cells. The results indicated that TBNPs effectively induced •OH generation in the mitochondria of tumor cells through Fenton reaction, with this effect markedly intensified upon laser irradiation. We hypothesize that the NPs decompose more rapidly under laser irradiation, leading to increased release of iron ions, which enhances the Fenton reaction. In contrast, no significant fluorescence levels were observed in the TCPP combined with laser group (Figure 3Q). These findings highlighted the effectiveness of the prepared TBNPs in generating ROS within the high‐GSH and high‐H₂O₂ tumor microenvironment. By leveraging both laser irradiation and the Fenton reaction, with the aid of TCPP, the NPs can induce significant oxidative stress and improve treatment outcomes.

### 2.6 Improved Cellular Uptake of TBNPs in Tumor Cells

**Figure 4:**
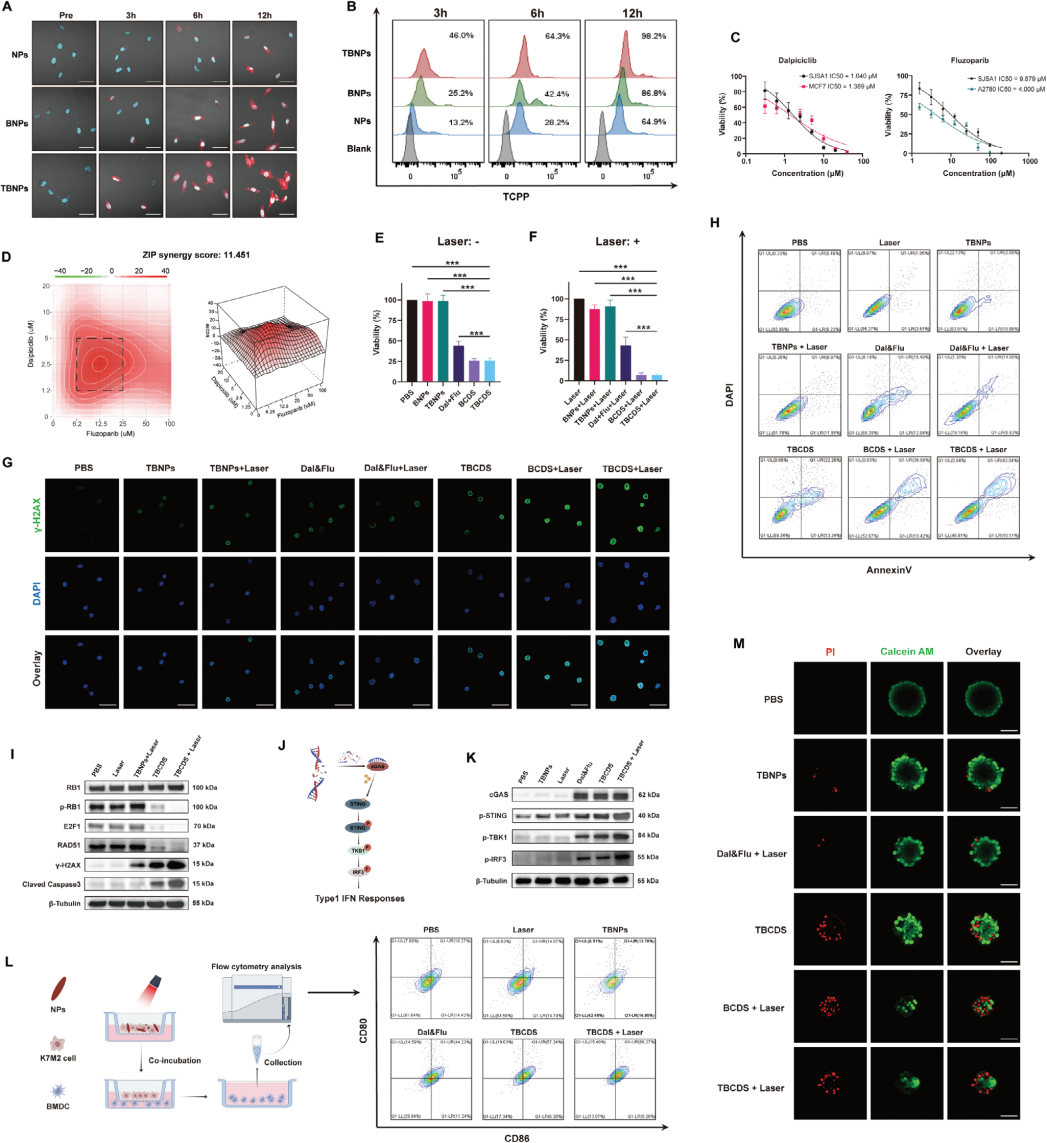
Efficacy of TBCDS‐Induced and Activation of cGAS‐STING. A) Representative CLSM images of cellular uptake of nanoparticles in tumor cells at different incubation time (n = 3; Scale bar = 50 µm). B) Flow cytometry analysis for osteosarcoma cells treated with TBNPs compared to NPs and BNPs (n = 3). C) Drug sensitivity tests for Dalpiciclib and Fluzoparib in SJSA1 cells (n = 6). D) Evaluation of synergistic effect for Dalpiciclib and Fluzoparib using SynergyFinder 3.0 (n = 3). E,F) Tumor cell inhibition comparison between TBCDS and other treatment groups (n = 6). G) Representative CLSM images of γ‐H2AX level in different treatment groups (n = 3; Scale bar = 50 µm). H) Flow cytometry analysis of Annexin V and DAPI staining tumor cells in different treatment (n = 3). I) Representative western blot analysis of p‐RB1, E2F1, RAD51, γ‐H2AX, and cleaved caspase3 in SJSA1 after different treatments (Combined with laser irradiation, n = 3). J) Schematic diagram of the activation of the cGAS‐STING signaling pathway. K) Representative western blot images of cGAS‐STING proteins in SJSA1 after different treatments, n = 3. L) Flow cytometry results of BMDCs maturation (CD11c^+^ CD80^+^ CD86^+^). M) Representative CLSM images of Live/Dead staining of 3D tumor spheroids after different treatments (n = 3; Scale bar = 100 µm). Results are all presented as the mean ± SD. The statistical analysis was performed using a one‐way ANOVA, *p < 0.05, **p < 0.01, ***p < 0.001.

To investigate the cellular uptake of the nanoparticles, fluorescence detection of TCPP was utilized. As illustrated in **Figure** **4**A, fluorescence emission at 660 nm was detected after 3 h of incubation with tumor cells, indicating these NPs have successfully entered tumor cells. Prolonged incubation further revealed an increased accumulation of TBNPs within the tumor cells, as evidenced by the enhanced fluorescence intensity. Flow cytometry analysis was performed to quantify and compare the uptake of nanoparticles at various time points. The results demonstrated that fluorescence levels were significantly higher in osteosarcoma cells treated with TBNPs compared to those treated with NPs or BNPs (Figure 4B). This differential fluorescence signal indicated that anti‐B7H3 targeting moiety was likely facilitating a more efficient binding and uptake of the TBNPs into the tumor cells, enhancing their selective delivery. Furthermore, the increased fluorescence over time underscores the potential of the TBNPs for sustained release and accumulation within the target cells, which could be advantageous for therapeutic applications.

### 2.7 Synergistic Effects of Dalpiciclib and Fluzoparib with Photodynamic Therapy

To assess the sensitivity of osteosarcoma cells to CDK4/6 inhibitors and PARP inhibitors, a CCK‐8 assay was utilized to evaluate cell proliferation. Drug sensitivity assays revealed IC50 values of 1.640 µM for Dalpiciclib and 9.879 µM for Fluzoparib in SJSA1 cells. Compared to the CDK4/6 inhibitor‐sensitive breast cancer cell line MCF7 (IC50 = 1.389 µM) and the PARP inhibitor‐sensitive ovarian cancer cell line A2780 (IC50 = 4.000 µM), SJSA1 cells exhibited relatively higher sensitivity (Figure 4C). To maximize the synergistic effects of the two drugs, SynergyFinder 3.0 was employed to quantify the synergy scores across various dose combinations.^[^ ^67^ ^]^ The analysis demonstrated that the two drugs exhibited synergistic effects over most of the dose range (synergy score > 0). Notably, the highest synergy score (11.451) was observed when Dalpiciclib and Fluzoparib were administered at a dose ratio of 1:5 (Figure 4D). Therefore, this dose ratio was employed to load the drugs into the NPs, thereby constructing BCDS or TBCDS for subsequent tumor treatment.

Cellular assays demonstrated that the BCDS group achieved superior tumor inhibition compared to dual‐drug treatment alone (Viability: BCDS vs Dal&Flu groups, 25.73 ± 2.60 vs 44.00 ± 5.61%). Additionally, the TBCDS group showed similar tumor suppression (Viability: TBCDS vs Dal&Flu groups, 25.82 ± 3.29 vs 44.00 ± 5.61%; *p* < 0.001; Figure 4E). When combined with PDT, both BNPs and TBNPs exhibited moderate tumor inhibition, whereas the dual‐drug group did not show significant changes (43.23 ± 10.08%). Notably, BCDS and TBCDS resulted in substantial tumor inhibition (BCDS group: 6.99 ± 2.52%, TBCDS group: 6.96 ± 1.47%), highlighting the enhanced therapeutic effect of combining PDT with the co‐delivery system (Figure 4F).

### 2.8 Activation of the cGAS‐STING Pathway and Maturation of Dendritic Cells by TBCDS

To assess the effects on DNA repair mechanisms, we conducted immunofluorescence detection of γ‐H2AX, a prominent marker for DNA damage.^[^ ^68^ ^]^ Significant DNA breaks were observed in both the TBNPs + Laser and Dal&Flu (Dalpiciclib + Fluzoparib) groups. Remarkably, the BCDS + Laser and TBCDS + Laser groups exhibited even higher levels of γ‐H2AX, indicating more extensive DNA damage (Figure 4G). Additionally, apoptosis analysis using Annexin V and DAPI staining demonstrated that Dal&Flu effectively induced apoptosis in tumor cells (12.63 ± 2.50%), while TBCDS resulted in an even greater proportion of apoptotic cells (24.17 ± 1.74%). Increased apoptosis level was further observed with the combination of PDT (42.46 ± 0.68%; TBCDS + Laser vs other groups: all *p* < 0.001; Figure 4H; Figure S13, Supporting Information). Next, western blot results revealed that the combination of Dalpiciclib and Fluzoparib reduced the expression of p‐RB1, its downstream target E2F1, and the homologous recombination protein RAD51. Both TBCDS and BCDS groups led to elevated levels of γ‐H2AX and cleaved Caspase 3 compared to the Dal&Flu group (Figure S14, Supporting Information). Notably, TBCDS, in combination with PDT, further increased γ‐H2AX and cleaved Caspase 3 levels, supporting the observed enhancement in DNA damage and apoptosis (Figure 4I).

Based on existing literature, DNA fragments generated from extensive DNA damage are known to activate the cGAS‐STING pathway^[^ ^69^ ^]^ (Figure 4J). Notably, CDK4/6 inhibitors and PARP inhibitors have been shown to activate cGAS‐STING pathway in colorectal cancer, thereby enhancing the immune microenvironment and sensitizing tumors to PD‐1 therapy.^[^ ^50^ ^]^ To explore this mechanism further, we investigated the activation of this pathway in the next step.

Western blot results indicated that both Dalpiciclib and Fluzoparib individually activated cGAS expression in SJSA1 cells and slightly increased the levels of p‐STING, p‐TBK1, and p‐IRF3. Importantly, when used in combination, the effect was significantly amplified, demonstrating their synergistic effect (Figure S15, Supporting Information). Considering that CDK4/6 inhibitors can upregulate the expression of PD‐L1 in tumor cells,^[^ ^70^, ^71^ ^]^ it is pertinent to explore whether B7H3, as a member of the same B7 family as PD‐L1, is also regulated by CDK4/6 inhibitors. Consistent with expectations, CDK4/6 inhibitors were found to upregulate the expression of B7H3, suggesting that the combination of CDK4/6 inhibitors with anti‐B7H3 therapy may hold potential for enhanced therapeutic efficacy (Figure S15, Supporting Information). In another set of WB for cGAS‐STING pathway, however, the TBCDS combined with PDT resulted in even higher activation level of the STING pathway, as evidenced by elevated levels of p‐STING, p‐TBK1, and p‐IRF3 compared to the Dal&Flu (Dalpiciclib + Fluzoparib) group, and the TBCDS group alone (Figure 4K). These findings indicated the possibility that DNA fragments resulting from substantial DNA damage effectively engage the cGAS‐STING pathway, thereby triggering the immune response against tumor cells.

To validate the impact of TBCDS on the phenotype of dendritic cells (DCs), K7M2 cells were first subjected to different treatments for 12 h in the upper chamber of a Transwell system. Following this treatment, these cells were co‐incubated with bone marrow‐derived dendritic cells (BMDCs) in the lower chamber. After the 12‐h treatment, the treated K7M2 cells were co‐cultured with BMDCs in the lower chamber for an additional 24 h. Subsequently, flow cytometry analysis was performed to assess the maturation status of BMDCs based on the expression of CD11c, CD80, and CD86. The results indicated that maturation rates of DCs in the PBS, TNPs, and Laser groups were relatively low (15.29 ± 1.05%, 14.20 ± 0.88%, and 13.27 ± 0.49%, respectively). However, treatment with Dal&Flu led to a visible increase in DC maturation (45.11 ± 1.06%), indicating the immunogenic potential of the combination therapy. TBCDS treatment significantly elevated the DC maturation rate to 54.69 ± 4.34%, highlighting the enhanced immune‐stimulating capacity of the co‐delivery system. Notably, TBCDS + Laser group demonstrated the highest DC maturation rate (65.98 ± 1.37%; TBCDS + Laser vs other groups: all *p* < 0.001). These results suggested that the combination of TBCDS with laser irradiation effectively promotes DC maturation through kill tumor cell, potentially enhancing anti‐tumor immune responses (Figure 4L; Figure S16, Supporting Information).

### 2.9 Superior Anti‐Tumor Activity of TBCDS in Clonogenic and 3D Spheroid Models

Clonogenic assays were conducted to evaluate the in vitro tumorigenic ability of SJSA1 cells after various treatments. The results, as shown in Figure S17 (Supporting Information), revealed that both the Dal&Flu and TBCDS groups effectively suppressed colony formation, highlighting their strong anti‐tumor properties. Notably, the TBCDS group exhibited a marked inhibition of colony formation under laser irradiation, indicating its potent anti‐tumor efficacy when combined with PDT. In contrast, the NPs combined with PDT did not show significant inhibition of colony formation, underscoring the superior effectiveness of DNA repair inhibitors in these conditions. To more accurately simulate the 3D structure and microenvironment of tumors, we employed 3D tumor spheroids for evaluating TBCDS efficacy. Unlike monolayer cultures, 3D spheroids provide a more realistic model that includes cell‐cell interactions and gradients of nutrients, oxygen, and drug concentrations, closely mimicking in vivo conditions.^[^ ^72^, ^73^ ^]^ Live/Dead staining was utilized to assess the viability of the tumor spheroids after treatment. The results showed that TBCDS treatment significantly reduced the size of the spheroids and increased the proportion of non‐viable cells after 72 h. The combination of PDT further enhanced these effects. In the TBCDS + Laser group, there were nearly no viable cells left, as indicated by the dramatic decrease in spheroid size compared to other groups (Figure 4M). This marked reduction in cell viability and spheroid size underscores the enhanced therapeutic efficacy achieved by targeting B7H3 in osteosarcoma treatment.

### 2.10 In Vivo Evaluation of Nanoparticle Biodistribution

**Figure 5:**
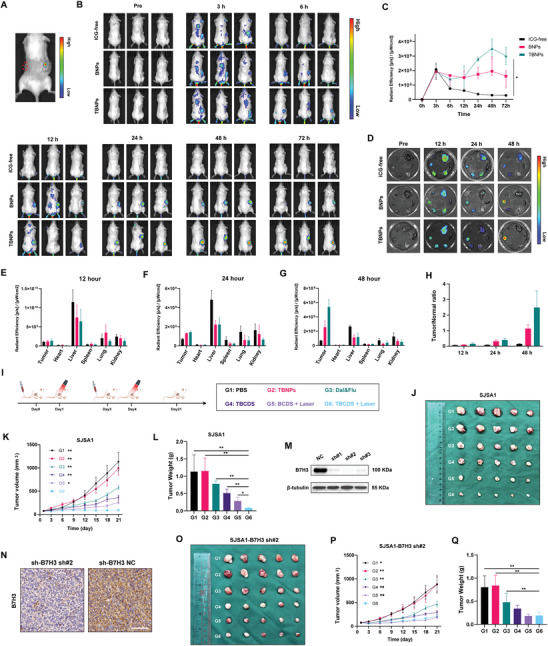
In Vivo Efficacy of TBCDS in Osteosarcoma‐Bearing Mice. A) In vivo imaging of TCPP fluorescence at the tumor site after intratumoral injection of nanoparticles. B) Biodistribution and metabolism of nanoparticles labeled ICG. C) Quantification of fluorescence intensity in tumor tissue at different time points. D) Representative Ex vivo fluorescence evaluation of tumors and major organs at 12, 24, and 48 h post‐injection, n = 3. E–G) Quantification of fluorescence intensity in tumor tissue and other vital organs at different time points, n = 3. H) Fluorescence intensity ratio between tumor tissues and vital organs at 12, 24, and 48 h, n = 3. I) Schematic diagram for the treatment regimens. J) Photographs of tumor tissues from different treatment groups at 21st day (SJSA1 xenograft tumor‐bearing NSG mice). K) Tumor volume measurements after different treatments (SJSA1 xenograft tumor‐bearing NSG mice, data are presented as the mean ± SEM; n = 5). L) Tumor weight measurements at 21st day after treatments (SJSA1 xenograft tumor‐bearing NSG mice, n = 5). M,N) Validation of SJSA1 cells with knocked‐down B7H3 expression (Scale bar = 100 µm). O) Photographs of tumor tissues from different treatment groups at 21st day (SJSA1‐B7H3 knockdown xenograft tumor‐bearing NSG mice). P) Tumor volume measurements after different treatments (SJSA1‐B7H3 knockdown xenograft tumor‐bearing NSG mice, data are presented as the mean ± SEM; n = 5). Q) Tumor weight measurements at 21st day after treatments (SJSA1‐B7H3 knockdown xenograft tumor‐bearing NSG mice, n = 5). Other data are all presented as the mean ± SD. The statistical analysis was performed with ANOVA analysis, *p < 0.05, **p < 0.01, ***p < 0.001.

To evaluate the GSH‐responsive ability of nanoparticles in vivo, nanoparticles were injected directly into the tumors of tumor‐bearing mice, and the fluorescence of TCPP at the injection site was monitored using an in vivo imaging system. As depicted in **Figure** **5**A, a distinct fluorescence signal was observed at the tumor site following intratumoral injection of nanoparticles, whereas no fluorescence was detected in nanoparticles injected subcutaneously on the contralateral side. This result indicated that the nanoparticles decompose in the high GSH environment of the tumor, leading to the release of TCPP.

For assessing the biodistribution and metabolism of nanoparticles, TNPs conjugated with ICG were administered via tail vein injection. Continuous monitoring of ICG fluorescence at various time points showed that ICG‐free had a short circulation time in the bloodstream, with negligible fluorescence observed beyond 12 h (Figure 5B). In contrast, BNPs and TBNPs, which are camouflaged with cell membrane components, effectively avoided macrophage phagocytosis, thereby extending their circulation time in the bloodstream. This prolonged circulation facilitated the accumulation of nanoparticles in tumor tissues via the Enhanced Permeability and Retention (EPR) effect. Notably, the nanoparticles accumulated significantly in tumor tissues, peaking at 48 h. Among them, TBNPs demonstrated a markedly higher fluorescence intensity in tumor tissues compared to BNPs (p = 0.200) and ICG‐free (p = 0.020), reflecting their enhanced tumor‐targeting capability (Figure 5C). In a separate experiment, mice were subsequently sacrificed at 12, 24, and 48 h post‐injection, and their tumors and major organs were harvested for ex vivo fluorescence evaluation (Figure 5D). Consistent with the in vivo imaging results, TBNPs exhibited a higher degree of accumulation in tumor tissues compared to BNPs and ICG‐free, with less accumulation observed in surrounding organs (Figure 5E–G). Additionally, the fluorescence intensity ratio between tumor tissues and surrounding organs indicated that TBNPs had significantly better tumor‐targeting capability in vivo compared to BNPs and ICG‐free (Figure 5H).

### 2.11 Anti‐B7H3 Improves Efficacy of TBCDS In Vivo

To further assess the in vivo efficacy of TBCDS combined with photodynamic therapy, a xenograft model was established using SJSA1 tumor‐bearing mice. Mice were randomly assigned into six groups: PBS, TBNPs, Dal&Flu, TBCDS, BCDS + Laser, and TBCDS + Laser, with treatment protocols outlined as shown in Figure 5I. Tumor volumes were measured every three days using calipers to evaluate antitumor effects. Consistent with previous findings, Dal&Flu exhibited antitumor activity and effectively inhibited tumor growth to a certain extent. The delivery of these drugs through TBNPs further enhanced their antitumor efficacy, although TBNPs alone did not lead to significant tumor suppression. Notably, the combination of TBCDS with PDT resulted in a marked improvement in tumor inhibition compared to the other treatment groups, including the BCDS + Laser group (p = 0.003; Figure 5J,K; Figure S18, Supporting Information). This enhanced effect was further corroborated by tumor weight measurements, which displayed a similar trend in tumor suppression (Figure 5L).

To confirm the role of anti‐B7H3 targeting in TBCDS, B7H3‐knockdown SJSA1 cells (SJSA1‐B7H3 shRNA) were generated (Figure 5M) and validated in tumor tissues of NSG mice bearing SJSA1 tumors (Figure 5N). Using the same treatment regimen and assessment methods, the TBCDS + Laser group continued to demonstrate significant antitumor effects compared to the other groups (PBS: p = 0.020, TBNPs: p = 0.010, and Dal&Flu: p = 0.009). However, no significant difference in tumor volume was observed between the TBCDS + Laser group and the non‐specifically targeted BCDS + Laser group (p = 0.730; Figure 5O,P; Figure S19, Supporting Information). Tumor weight measurements corroborated this observation (Figure 5Q). Additionally, there were no notable fluctuations in body weight across the various treatment groups (Figures S18 and S19, Supporting Information). These results indicate that although TBCDS combined with PDT offers substantial antitumor benefits, the specific targeting B7H3 is crucial for achieving maximum therapeutic efficacy in osteosarcoma treatment.

### 2.12 Combination TBCDS with PDT Boosts CD8^+^ T Cell Infiltration in Tumor Microenvironment

**Figure 6:**
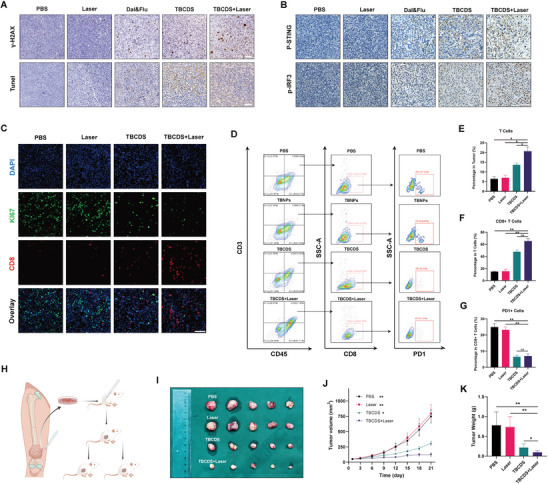
Evaluation of TBCDS combined with PDT in osteosarcoma models. A) Representative images of γ‐H2AX and Tunnel staining in tumor tissues of K7M2 tumor‐bearing BALB/c mice (n = 5; Scale bar = 100 µm). B) Representative images of p‐STING and p‐IRF3 staining in tumor tissues of K7M2 tumor‐bearing BALB/c mice (n = 5; Scale bar = 50 µm). C) Representative immunofluorescence staining for Ki67, and CD8 in K7M2 tumor tissues of BALB/c mice after different treatments (n = 3; Scale bar = 50 µm). D) Flow cytometry analysis of immune cell infiltration in tumors after different treatments (n = 3). E–G) Statistical analysis of tumor‐infiltrating T cells, CD8+ T cells, and PD1+ CD8+ T cells across different treatment groups (n = 3). H) Schematic of the PDX model experimental protocol. I) Photographs of PDX tumor tissues on the 21st day. J) Tumor volume measurements after different treatments in PDX models (Data are presented as the mean ± SEM; n = 5). K) Tumor weight measurements at 21st day in PDX models (Data are presented as the mean ± SD; n = 5). The statistical analysis was performed with ANOVA analysis, *p < 0.05, **p < 0.01, ***p < 0.001.

To investigate the effects of TBCDS on the tumor immune microenvironment, particularly through its activation of the cGAS‐STING pathway via extensive DNA strand breaks, BALB/c mice bearing K7M2 murine osteosarcoma tumors were utilized. Immunohistochemical analysis of K7M2‐derived tumor tissues confirmed high expression of B7H3 on the tumor cell membranes (Figure S20, Supporting Information), consistent with prior findings. Notably, human B7H3, a type I transmembrane glycoprotein, has two isoforms: 2IgB7‐H3 and 4IgB7‐H3. Mouse B7H3, structurally analogous to human 2IgB7‐H3,^[^ ^74^ ^]^ shares 93% amino acid similarity, thus theoretically enabling effective targeting of K7M2 cells by TBCDS.^[^ ^22^ ^]^ First, immunohistochemical staining for γ‐H2AX and TUNEL was conducted to assess DNA damage and apoptosis in K7M2 tumor‐bearing BALB/c mice treated with different groups. As illustrated in **Figure** **6**A, the Dal&Flu (Dalpiciclib and Fluzoparib) treatment group exhibited significantly enlarged tumor cells, suggestive of senescence, which aligns with the mechanism of CDK4/6 inhibition. Moreover, increased expression levels of γ‐H2AX and TUNEL were observed in the Dal&Flu group compared to the PBS and Laser groups. The delivery of TBNPs further augmented γ‐H2AX and TUNEL expression levels in TBCDS group. Crucially, when tumors were subjected to TBCDS combined with photodynamic therapy, a marked elevation in γ‐H2AX expression was noted, indicating substantial DNA damage induced by this therapeutic approach.

Based on the hypothesis that DNA breakage leads to the activation of the cGAS‐STING pathway, p‐STING, and p‐IRF3 were further assessed to verify this pathway's activation. As shown in Figure 6B and p‐IRF3 and p‐STING exhibited a consistent expression pattern. Notably, mild activation of the STING pathway was observed in Dal&Flu group, albeit at lower levels. In contrast, the TBCDS group combined with PDT demonstrated the most pronounced activation of the STING pathway compared to other treatment groups. Similarly, WB results from mouse tumor tissues corroborated the activation of the STING pathway in the TBCDS related treatment, as evidenced by increased levels of cGAS, p‐STING, p‐TBK1, and p‐IRF3 (Figure S21, Supporting Information). These findings suggested potential alterations in the osteosarcoma tumor microenvironment (TME) under TBCDS treatment.

To further evaluate alterations in the immune microenvironment within murine tumor tissues, immunofluorescence staining was performed. The results revealed that tumors treated with TBCDS exhibited a higher infiltration of CD8^+^ T cells and a marked reduction in Ki67^+^ proliferative cells compared to the PBS control group, indicating that TBCDS effectively inhibited tumor cell proliferation while activating the immune microenvironment (Figure 6C). In contrast, laser irradiation alone did not significantly inhibit tumor growth or enhance CD8^+^ T cell infiltration. However, when combined with TBCDS, a further reduction in Ki67^+^ cells and a significant increase in CD8^+^ T cell infiltration were observed. To quantify the immune activation, multi‐color flow cytometry was employed to evaluate the proportion of CD8^+^ T cells across the four treatment groups (PBS, TBNPs, TBCDS, and TBCDS + Laser) (Figure 6D). The results indicated that the PBS group exhibited a low proportion of infiltrating T cells (6.42 ± 1.18%) and a high proportion of exhausted T cells (PD1^+^CD8^+^ T cells; 24.92 ± 2.07%), consistent with the immunologically “cold” feature of osteosarcoma. In the TBCDS‐treated group, there was a significant increase in the proportion of infiltrating T cells (13.68 ± 0.90%) and CD8^+^ T cells (47.77 ± 2.75%), accompanied by a substantial decrease in the proportion of PD1^+^CD8^+^ T cells (6.51 ± 0.89%). Notably, the TBCDS + Laser group achieved the highest levels of overall T cell infiltration (20.70 ± 2.21%) and CD8^+^ T cell infiltration (65.34 ± 4.22%) among all groups, alongside a pronounced reduction in PD1^+^CD8^+^ T cells (6.98 ± 1.39%) (Figure 6E–G). These findings suggested that the combination of TBCDS with PDT not only enhances T cell infiltration within the tumor microenvironment but also mitigates T cell exhaustion, potentially facilitating a more robust antitumor immune response.

Previous studies have shown that the osteosarcoma microenvironment comprises not only T cells but also various other cell types, including macrophages, endothelial cells, and fibroblasts.^[^ ^33^, ^75^ ^]^ Therefore, the potential of TBCDS to induce changes in these immune cell populations necessitates further investigation. Although B7H3 is widely recognized as an immunomodulatory molecule, several studies have demonstrated that targeting B7H3 can enhance CD8+ T cell infiltration.^[^ ^76^ ^]^ However, in the context of sarcomas, which are typically considered immunologically “cold” tumors, evidence remains limited as to whether targeting B7H3 could induce widespread immune cell alterations within the TME. To exclude the potential impact of B7H3 on the cGAS‐STING pathway, the expression of key factors related to the STING pathway was further evaluated in both B7H3 knockdown SJSA1 cells and control SJSA1 cells. Western blot analysis indicated no significant differences in the levels of STING pathway markers between control and B7H3 knockdown cell lines (Figure S22, Supporting Information), indicating that B7H3 knockdown does not affect the cGAS‐STING pathway in SJSA1 cells.

Consequently, it is proposed that the observed mchanges in the tumor microenvironment are primarily driven by the payloads of the delivery system, namely, CDK4/6 inhibitors and PARP inhibitors. This hypothesis is grounded not only in their established anti‐tumor mechanisms but also in their potential to modulate the immune microenvironment when used independently. PARP inhibitors, as classic DNA repair inhibitors, primarily enhance immune responses by activating the cGAS‐STING pathway,^[^ ^77^ ^]^ which leads to increased maturation of dendritic cells and infiltration of CD8^+^ T cells. However, comprehensive evidence regarding the broader impact of PARP inhibitors on other cell types within the microenvironment remains limited and inconsistent. In contrast, CDK4/6 inhibitors exhibit multifaceted anti‐tumor effects through mechanisms that include cell cycle arrest, metabolic regulation, and induction of senescence. Their impact on the TME is complex, extending beyond mere activation of the STING pathway.^[^ ^78^ ^]^ CDK4/6 inhibitors have been demonstrated to prevent T cell exhaustion by inhibiting cell cycle progression, thereby preserving T cell survival and functionality, which enhances anti‐tumor immune responses.^[^ ^79^ ^]^ Additionally, CDK4/6 inhibitors can reduce regulatory T cell infiltration and inhibit immunosuppressive pathways, further potentiating anti‐tumor immunity.^[^ ^80^ ^]^ Additionally, CDK4/6 inhibitors can reduce the infiltration of regulatory T cells and inhibit immunosuppressive pathways, further potentiating anti‐tumor immunity.^[^ ^81^ ^]^ The findings of this study demonstrated significant alterations in T cells within the immune microenvironment mediated by TBCDS, which may be attributed to the immune “cold” nature of osteosarcoma. Nevertheless, the effects of TBCDS on other cellular components within the TME necessitate further investigation through immunological experiments in future studies.

### 2.13 TBCDS Combined with PDT Significantly Suppresses Osteosarcoma Growth in PDX Model

To facilitate clinical translation, a patient‐derived xenograft (PDX) model of osteosarcoma was established to evaluate the efficacy of TBCDS combined with PDT (Figure 6H). PDX models are considered one of the best preclinical models available, as they preserve the genetic and histological characteristics of the original human tumors, providing a more accurate representation of therapeutic efficacy.^[^ ^82^ ^]^ Consistent with previous findings, treatment with TBCDS alone resulted in a moderate inhibition of tumor growth. However, when TBCDS was combined with PDT, there was a significant suppression of PDX tumor growth compared to other groups (Figure 6I,J; Figure S23, Supporting Information). The changes in body weight among the mice were negligible, indicating that the NPs exhibited minimal systemic toxicity (Figure S23, Supporting Information). The tumor weight measurements further corroborated these findings (PBS: 0.78 ± 0.34 g, Laser:0.74 ± 0.26 g, TBCDS: 0.22 ± 0.09 g, TBCDS + Laser: 0.06 ± 0.02 g; p < 0.01) (Figure 6K), indicating that TBCDS, designed based on the genomic information of osteosarcoma, in combination with PDT, can effectively inhibit tumor growth in the PDX model.

Considering that osteosarcoma encompasses multiple histological subtypes, each characterized by significant differences in prognosis and therapeutic response, this presents a substantial challenge in the treatment of osteosarcoma.^[^ ^83^ ^]^ In contrast, the constructed TBCDS adopts a multi‐dimensional anti‐tumor strategy rather than relying on a single target. As originally designed, the delivery system incorporates CDK4/6 inhibitors and PARP inhibitors, which exhibit complementary sensitization mechanisms. Furthermore, the expression rate of B7H3 exceeds 90% in osteosarcoma tissues, ensuring the targeted delivery capability of this system. Building on these foundations, the hydroxyl radicals generated by the Fe‐TCPP material demonstrate robust and broad‐spectrum tumoricidal effects. This not only enhances the therapeutic ceiling of the co‐delivery system but also strengthens its efficacy baseline, thereby allowing TBCDS to maintain anti‐tumor activity independent of specific tissue subtypes. In future work, it is essential to further validate the efficacy of TBCDS across various histological subtypes of osteosarcoma. This could be accomplished by establishing primary cell models or PDX models from diverse subtypes for both in vitro and in vivo evaluations. Additionally, conducting prospective clinical trials will be necessary to substantiate the efficacy of TBCDS.

### 2.14 Safety Evaluation of Nanoparticles

To evaluate the safety of the NPs, a series of tests were conducted. First, a hemolysis assay was performed to assess hemocompatibility at various concentrations. The results showed a dose‐dependent hemolytic activity, with higher concentrations causing increased hemolysis. Notably, at concentrations below 5 mg mL^−1^, the hemolysis percentage remained below 5%, indicating acceptable hemocompatibility at normal doses (Figure S24A,B, Supporting Information). Next, blood routine and biochemical analyses were carried out for different treatment groups, including PBS, Laser, TBNPs, Dal&Flu, TBCDS, and TBCDS + Laser. The measured parameters included red blood cells (RBC), white blood cells (WBC), hemoglobin (HGB), platelets (PLT), alanine aminotransferase (ALT), and aspartate aminotransferase (AST). The results showed no significant abnormalities across the treatment groups, suggesting that the nanoparticles and treatments did not induce systemic toxicity in the mice (Figure S24C, Supporting Information). Importantly, hematoxylin and eosin (HE) staining of vital organs (heart, lung, liver, and spleen) was performed to assess any histopathological changes. Microscopic analysis revealed no significant tissue damage or pathological alterations in any of the treatment groups compared to the control, further supporting the biocompatibility and safety of the nanoparticles (Figure S24D, Supporting Information).

## 3 Conclusion 

In this study, bioinformatics analysis was employed to identify potential target molecules in osteosarcoma, revealing its genomic characteristics that suggest potential sensitivity to combination therapy with CDK4/6 inhibitors and PARP inhibitors. Building on these insights, a biomimetic co‐delivery system targeting B7H3 was developed. This system incorporates a GSH‐responsive function, enabling the controlled release of drugs and TCPP within tumor. In both in vivo and in vitro experiments, TBCDS demonstrated significant tumor‐suppressive effects, notably increasing DNA damage and cell apoptosis, particularly when combined with PDT. Moreover, the combination of TBCDS with PDT effectively enhanced CD8^+^ T cell infiltration, transforming the osteosarcoma immune microenvironment from an immunologically “cold” to a “hot” state. In conclusion, TBCDS combined with PDT represents an effective and safe therapeutic strategy, holding considerable promise as a novel approach for the treatment of osteosarcoma.

## Conflict of Interest

The authors declare conflict of interest.

## Author Contributions

T.L., Z.F., and A.Z. contributed equally to this work. T.L. performed conceptualization, validation, and writing. Z.F. performed methodology, investigation, and writing. A.Z. performed visualization, validation, and writing. A.W. performed investigation and writing. Y.P. performed validation and writing. Y.X. performed validation. D.N., H.C. and W.C. performed software. J.L. and M.G. performed the methodology. A.H. performed formal analysis. Y.H. performed data curation. Y.D. performed data curation. X.Z. performed funding acquisition and project administration. L.R. wrote, reviewed, and manages project administration. J.W. performed conceptualization, funding acquisition, and Supervision.

## Supporting information



Supporting Information

## Data Availability

The data that support the findings of this study are available from the corresponding author upon reasonable request.
