# Supplementary material for: Biomimetic Targeted Co‐Delivery System Engineered from Genomic Insights for Precision Treatment of Osteosarcoma
Source: Adv Sci (Weinh). 2024 Nov 18;12(2):2410427. doi: 10.1002/advs.202410427 (PMC11727257; doi:10.1002/advs.202410427)
Supplement: Supplementary file 1 — Supporting Information [file ADVS-12-2410427-s001.docx]

*Supporting Information*

**Biomimetic Targeted Co-Delivery System Engineered from Genomic Insights for Precision Treatment of Osteosarcoma**

*Tianqi Luo^†^, Zhijin Fan^†^, Anyu Zeng^†^, Anqi Wang, Yuanwei Pan, Yanyang Xu, Hongmin Chen, Weiqing Chen, Dingmeng Nie, Jiaming Lin, Anfei Huang, Ming Gong, Yufeng Huang, Yun Ding, Xiaojun Zhu*, Lang Rao*, Jin Wang**

T. Luo, A. Zeng, Dr. A. Wang, Dr. H. Chen, W. Chen, Dr. J. Lin, Dr. A. Huang, Dr. Y. Huang, Prof. X. Zhu, and Prof. J. Wang

Department of Musculoskeletal Oncology, State Key Laboratory of Oncology in South China, Sun Yat-Sen University Cancer Center, Guangzhou 510060, China.

Email: zhuxj@sysucc.org.cn; wangjinr@sysucc.org.cn

D. Nie*,* Prof. Y. Pan and Prof. L. Rao

Institute of Chemical Biology, Shenzhen Bay Laboratory, Shenzhen 518132, China.

Email: lrao@szbl.ac.cn

Prof. Z. Fan

Institute for Engineering Medicine,

Kunming Medical University, Kunming 650500, China.

Dr. Y. Xu

Department of Joint Surgery, Guangzhou First People’s Hospital, School of Medicine, South China University of Technology, Guangzhou 510060, China.

Dr. M. Gong

Department of Pediatric Orthopaedics, Guangzhou Women and Children’s Medical Center, Guangzhou Medical University, Guangdong Provincial Clinical Research Center for Child Health, Guangzhou 510630, China.

Y. Ding

Department of Breast Oncology, State Key Laboratory of Oncology in South China,

Sun Yat-sen University Cancer Center, Guangzhou 510060, China.

^†^ These authors contributed equally: Tianqi Luo, Zhijin Fan, Anyu Zeng.

1. **Method**
   1. **Materials and Reagents.**

The materials used for the synthesis of nanoparticles include 4,4,4-(Porphine-5,10,15,20-tetrayl) tetrayl (TCPP, Cat #P816654), FeCl3 6H2O (Cat # I809489), and 2-Aminoterephthalic acid (Cat # BD00739675). Antibodies for molecular biology experiment included γ-H2A.X (phospho S139) (Cat #ab81299), RAD51 (Cat #ab133534), E2F1 (Cat #ab314311), RB1 (Cat #ab181616), Phospho-Rb (Ser807/811) (Cat #8516), and Cleaved Caspase-3 (Cat #9661), Human B7H3 (Cat #ab227679), mouse B7H3 (Cat #AG5226), cGAS (Cat #9661; Cat #79978), Phospho-TBK1/NAK (Ser172) (Cat #5483), Phospho-IRF3 (Ser396) (Cat # AF2436), Phospho-STING (Ser366) (Cat #AF7416), GAPDH (Cat #AF7021) and β-Tubulin (Cat #2128). For flow cytometry analysis, the following antibodies and dyes were used: APC anti-B7H3 (Cat #ab275659), Anti-Myc tag (Cat #ab117487), anti-mouse CD16/CD32 (Cat #553141), Fixable Viability Dye (Cat #65-0863-14), anti-mouse CD3 FITC (Cat #561798), anti-mouse CD45 RB705 (Cat #570291), anti-mouse CD8a PE-Cy7 (Cat #552877), anti-mouse PD-1 BV421 (Cat #135221), CD11c-FITC (Cat #), CD80-APC (Cat #), and CD86-PE (Cat #). Other reagents include Dalpiciclib (Cat #HY-114338), Fluzoparib (Cat # HY-114778), ICG (Cat #I811816), DCFH-DA (Cat #G2716-1), SOSG (Cat #MA0326), MitoROS^TM^OH580 (Cat # 16055), TMB (Cat #T818987), CalceinAM (Cat #C2012), pHrodo (Cat #P36600), Hoechst33342 (Cat #C1028), DAPI (Cat #C1005), DIO (Cat #D4007), PI (Cat #Cat #AC10860), Annexin V (Cat #AC10860), and Cell Counting Kit-8 (Cat # C0005).

- 1. **Engineering of RAW 264.7 Cells**

The anti-B7H3 protein sequence, including a MYC-tag, hinge region, and transmembrane region, was added into a lentiviral vector. This vector was co-transfected with packaging plasmids into HEK293T cells using Lipofectamine 3000. Forty-eight hours post-transfection, lentiviral particles were collected and concentrated by ultracentrifugation. The concentrated viral particles were used to infect RAW 264.7 cells in the presence of polybrene (8 μg/mL). After 24 hours, the medium was replaced with fresh complete DMEM. To validate the presence of the anti-B7H3 protein on the cell membrane, flow cytometry and immunofluorescence were performed using anti-MYC-tag antibodies. Western blot analysis was conducted to further confirm the expression of the anti-B7H3 protein.

- 1. **Bioinformatics Analysis**

The expression of B7H3 across thirty-three tumor types was analyzed using TCGA database (https://portal.gdc.cancer.gov/). Genomic mutations in the MSKCC-OS and TCGA-SARC cohorts were examined through cBioPortal (https://www.cbioportal.org/). Single-cell RNA sequencing data from GSE162454 (https://www.ncbi.nlm.nih.gov/geo/) were analyzed using R package “Seurat” for clustering and differential expression. Pseudotime trajectory analysis was conducted with R package “Monocle”, and cell communication networks were assessed using R package “CellChat”. Genomic instability and HRD scores were calculated with R package “scarHRD”, while GSEA was performed using R package “clusterProfiler”. Survival analyses for CDK4 and HRDscore were conducted with R package “survival”. Protein structure prediction and molecular docking analysis were performed using AlphaFold3 (https://alphafoldserver.com/) and ChimeraX (1.8 version), respectively.

- 1. **Preparation of TBCDS**

Fe-MOF nanoparticles were prepared by dissolving TCPP (0.1 g), 2-Aminoterephthalic acid (BDC-NH2, 0.2264 g) and ferric chloride hexahydrate (FeCl₃·6H₂O, 2.7 g) in 150 mL of N, N-Dimethylformamide (DMF). The solution was then stirred at 90°C for 6 hours. The resulting precipitate (Fe-MOF) was collected by centrifugation, washed and stored in ethanol. Then, Fe-MOF (30 mg) was dissolved in deionized water (10 mL) and rotated with a dimethyl sulfoxide (DMSO, 1 mL) suspension of Dalpiciclib (2 mg) and Fluzoparib (10 mg). After stirring for 12 h at room temperature, the Fe-MOF loaded with Dalpiciclib and Fluzoparib (Co-delivery system) was collected by centrifugation and washed twice with deionized water. Next, the prepared anti-B7H3 RAW membrane was ultrasonically mixed with the Co-delivery system for 5 min, and centrifugated at 4°C. After washing, the collected TBCDS were suspended in deionized water by ultrasound and stored at 4°C.

- 1. **Characterization of Nanoparticles**

The particle size and zeta potential were measured using dynamic light scattering (DLS) with a Zetasizer Nano ZS (Malvern Instruments, MA, USA), with nanoparticles dispersed in deionized water at 10 μg/mL and sonicated prior to measurement. Morphological analysis was performed with transmission electron microscopy by placing a nanoparticle suspension on a carbon-coated copper grid and imaging with a TEM at 200 kV. HPLC was employed to determine the encapsulation efficiency and drug loading capacity of the NPs. Encapsulation efficiency and drug loading capacity were calculated using the following formulas:

*Encapsulation* *Efficiency (%) = (mass of the Dal or Flu in feed - Dal or Flu mass removed by centrifugation) / (mass of the Dal or Flu in feed) × 100 %*;

*Loading Efficiency (%) = (mass of the Dal or Flu in feed - Dal or Flu mass removed by centrifugation) / (the total mass of MOFs and Dal or Flu in feed) × 100 %*;

Elemental analysis of NPs was conducted using energy-dispersive X-ray spectroscopy (EDS) with a scanning electron microscope equipped with an EDS detector. X-ray photoelectron spectroscopy (XPS) was used to analyze the surface composition and chemical states, with spectra obtained from an AXIS Ultra DLD spectrometer using an Al Kα X-ray source. The •OH and ^1^O_2_ were captured using 5′5-dimethyl-1-pyrroline N-oxide (DMPO) and 2,2,6,6-tetramethylpiperidine (TEMP), respectively, resulting in the formation of moderately stable adducts for detection purposes.

- 1. **Cell Culture**

The murine osteosarcoma cell line K7M2 and mouse macrophage cell line RAW 264.7 were cultured in Dulbecco's Modified Eagle Medium (DMEM) supplemented with 10% fetal bovine serum (FBS), 100 U/mL penicillin, and 100 μg/mL streptomycin. The human osteosarcoma cell line SJSA1 was cultured in RPMI 1640 medium supplemented with 10% FBS, 100 U/mL penicillin, and 100 μg/mL streptomycin. All cells were maintained at 37°C in a humidified atmosphere containing 5% CO₂. For B7H3 shRNA construction, B7H3-specific shRNA sequences were designed and cloned into a lentiviral vector. The lentiviruses were produced by transfecting HEK293T cells with the shRNA-containing vector along with the packaging plasmids using Lipofectamine 3000. After 48 hours, the viral supernatant was collected, filtered, and used to transduce target SJSA1. Following transduction, cells were selected with 2 µg/mL puromycin for 7 days to establish stable B7H3 knockdown cell lines.

- 1. **In Vitro Experiments**

For cell uptake, SJSA1 cells were seeded into confocal dishes for 24 h, and nanoparticles (NPs, BNPs and TBNPs; 50 μg/mL) were incubated for 0, 3, 6, and 12 h. After being washed, the cells were detected using confocal microscope. For ROS detection, SJSA1 cells were pre-seeded into confocal culture dishes to allow for proper adherence and growth. Following seeding, the cells were incubated with TCPP (25 μg/mL) or different groups of nanoparticles (NPs, BNPs and TBNPs; 50 μg/mL) for 3 hours. For groups involving laser irradiation, the cells were exposed to laser (660 nm, 100 mW cm^−2^) for 5 minutes. Next, the cells were treated with various ROS-specific probes: DCFH-DA for detecting general intracellular ROS, SOSG for identifying singlet oxygen, and MitoROS^TM^OH580 for specifically measuring mitochondrial hydroxyl radicals. For cell proliferation assay, SJSA1 cells were seeded into 96-well plates at a density of 2,000 cells per well in 100 µL of culture medium and allowed to adhere overnight. The next day, after refreshing the medium, cells were treated according to the different treatment protocols: PBS, Dal&Flu (Dalpiciclib 2.5 μM + Fluzoparib 12.5 μM), and nanoparticles (42 μg/mL; BNPs, TBNPs, BCDS, and TBCDS). For groups involving laser irradiation, the cells were exposed to laser (660 nm, 100 mW cm^−2^) for 5 minutes at 3 hours after drug administration. After 72 hours of treatment, the medium was replaced with fresh culture medium containing 10% Cell Counting Kit-8 (CCK-8) reagent in each well. The cells were incubated at 37°C for an additional 1-4 hours. Cell viability was then assessed by measuring the absorbance at 450 nm using a microplate reader. For cellular immunofluorescence detection, 1 × 10⁵ SJSA1 cells in 1 mL of culture medium were seeded into confocal dishes and allowed to adhere overnight. Subsequently, after 48 hours of treatment with the specified methods, the cells were examined. For colony formation assay, 500 SJSA1 cells in 1 mL of culture medium were seeded into 12-well plates at a density of 500 cells per well and allowed to adhere overnight. The following day, treatments were applied as per the experimental design. Cells were then incubated for 10 days to allow colony formation. After the incubation period, colonies were fixed with 4% paraformaldehyde for 15 minutes and stained with 0.1% crystal violet for 30 minutes. The stained colonies were washed with PBS, air-dried, and photographed for analysis.

- 1. **Isolation of Bone Marrow-derived Dendritic Cells and Co-Culture with Tumor Cells**

Bone marrow-derived dendritic cells (BMDCs) were isolated from the femur and tibia of 6–8-week-old Balb/c mice. Briefly, the bone marrow was flushed with sterile PBS, and the collected cells were passed through a 70 µm cell strainer to obtain a single-cell suspension. The cells were then centrifuged at 300 g for 5 minutes and resuspended in RPMI-1640 medium supplemented with 10% FBS, 20 ng/mL GM-CSF, and 10 ng/mL IL-4. The cell suspension was cultured for 7 days, with a medium change every 3 days to allow for the differentiation and maturation of BMDCs. On day 7, non-adherent cells were collected, washed, and used for further experiments. For the co-culture experiments, K7M2 osteosarcoma cells were first seeded into the upper chamber of a Transwell system and subjected to various treatments for 12 hours. Subsequently, the upper chamber containing the treated K7M2 cells was placed over a lower chamber containing BMDCs. The co-culture was continued for 24 hours to allow interaction between the treated K7M2 cells and BMDCs. After co-incubation, the BMDCs were collected for flow cytometric analysis to evaluate their maturation levels by assessing CD11c, CD80 and CD86 expression.

- 1. **Tumor Spheroids**

Tumor spheroids were cultured in ultra-low attachment 96-well plates. When the spheroids reached a diameter of approximately 100 μm, these spheroids were seeded into ultra-low attachment 6-well plates. Then, treatment was initiated according to the previous experimental protocol. After 72 hours of treatment, a Live/Dead staining assay was performed to assess cell viability within the spheroids. The staining solution was prepared by diluting Calcein-AM (final concentration 2 µM) and propidium iodide (final concentration 4 µM) in medium. The spheroids were incubated with the staining solution at 37°C for 30 minutes. Fluorescence imaging was conducted using a confocal microscope. Calcein-AM was excited at 488 nm, and its fluorescence emission was collected between 500-530 nm to identify live cells (green fluorescence). Propidium iodide was excited at 543 nm, and its fluorescence emission was collected between 610-650 nm to identify dead cells (red fluorescence). Images were captured and analyzed to determine the viability of cells within the spheroids and to evaluate the size of the spheroids.

- 1. **Flow Cytometry**

After the SJSA1 cells were treated according to the previous experimental protocol, cells were harvested, washed with PBS, and resuspended in binding buffer at a concentration of 1×10⁶ cells/mL. For Annexin V and DAPI staining, 5 µL of Annexin V-FITC and 5 µL of DAPI were added to 100 µL of the cell suspension. The mixture was gently vortexed and incubated for 15 minutes at room temperature in the dark. After incubation, 400 µL of binding buffer was added to each sample. Flow cytometry was performed immediately. To assess changes in the tumor immune microenvironment, fresh tumors are collected and enzymatically dissociated using collagenase and DNase I, then filtered and centrifuged to obtain a single-cell suspension. After cell counting, the suspension is blocked with an anti-CD16/CD32 Fc receptor antibody to prevent non-specific binding. The cells are stained with a panel of antibodies, including Dead APC-CY7, CD3-FITC, CD45-PerCP-CY5.5, CD8-PE-CY7, and PD1-BV421. Following incubation with these antibodies, the cells were washed twice with PBS containing 1% bovine serum albumin (BSA) and resuspended in PBS for flow cytometry analysis.

- 1. **Animal Models**

Six-week-old female immunodeficient NSG (NOD/SCID/IL2Rγnull) mice, obtained from the Sun Yat-sen University Cancer Center Animal Experiment Center, were used to establish SJSA1 and Patient-derived xenograft (PDX) models. For the SJSA1 xenograft model, 1×10⁶ SJSA1 cells were suspended in 100 µL of PBS containing 50% Matrigel and injected subcutaneously into the right flank of each mouse. The same experimental protocol was also applied to establish BALB/c mice bearing K7M2 tumors to evaluate changes in the immune microenvironment and assess safety. For the PDX models, osteosarcoma patient’s tumor tissue was cut into small fragments (2-3 mm³) under sterile conditions and similarly implanted subcutaneously into the right flank of the mice. This study has been approved by the Animal Ethics Committee of the SYSUCC (L025501202211020).

- 1. **Distribution and Antitumor Efficacy of Nanoparticles in Vivo**

The NPs were labeled with ICG to enable non-invasive imaging using an in vivo imaging system (IVIS). ICG-free, BNPs, and TBNPs (5 mg/mL, 100 μL) were injected into the tail vein. Then, mice were imaged at various time points post-injection to monitor the biodistribution and tumor accumulation of the nanoparticles. The fluorescence intensity (Ex: 740 nm, Em:800 nm) within the tumor and major organs was quantified to evaluate the targeting efficiency and distribution pattern of the NPs. For evaluation of antitumor efficacy, once the tumors reached approximately 50 mm³ in volume, the mice were randomly divided into different treatment groups: PBS (control), Laser, NPs (TBNPs, BCDS, TBCDS 100 μL, 10 mg/mL in PBS), and TBCDS + Laser. Laser irradiation was performed using a laser (wavelength: 660 nm) at a power density of 200 mW/cm² for 5 minutes. For unencapsulated Dal&Flu group, Dalpiciclib (25 μg) and Fluzoparib (125 μg) were suspended in a 100ul PBS and injected intraperitoneally for each mouse. Tumor volume was measured every three days using calipers, and the volume was calculated using the formula: *Volume = (length × width^2^)/2*. Tumor weight was recorded at the end of the treatment period.

- 1. **Immunohistochemistry and Immunofluorescence Staining**

Tumor tissues were fixed in 4% paraformaldehyde, embedded in paraffin, and sectioned into 5 µm thick slices. For IHC staining, sections were deparaffinized, rehydrated, and subjected to antigen retrieval by heating in a citrate buffer (pH 6.0) for 20 minutes. Endogenous peroxidase activity was blocked using 3% hydrogen peroxide for 10 minutes. Sections were then incubated with primary antibodies against γ-H2AX, TUNEL, B7H3, p-STING and p-IRF3 overnight at 4°C. After washing, sections were incubated with appropriate secondary antibodies conjugated to horseradish peroxidase (HRP) for 1 hour at room temperature. Visualization was achieved using diaminobenzidine (DAB) as the chromogen, followed by counterstaining with hematoxylin.

For cell immunofluorescence, cells were cultured on confocal dishes and fixed with 4% paraformaldehyde for 15 minutes at room temperature. After washing with PBS, the cells were permeabilized with 0.1% Triton X-100 for 10 minutes. Blocking was performed using 5% BSA in PBS for 1 hour. The cells were then incubated overnight at 4°C with a primary antibody. After washing with PBS, the cells were incubated with a fluorescently labeled secondary antibody for 1 hour at room temperature in the dark. Finally, fluorescence images were captured using a confocal microscope. For tissues immunofluorescence staining, tumor tissue sections were prepared similarly to IHC. After antigen retrieval, sections were blocked with 5% bovine serum albumin (BSA) for 1 hour at room temperature. Primary antibodies against Ki67 and CD8 were applied and incubated overnight at 4°C. The next day, sections were washed and incubated with fluorophore-conjugated secondary antibodies for 1 hour at room temperature in the dark. Nuclei were counterstained with DAPI for 5 minutes. Sections were then mounted with antifade mounting medium and visualized using a confocal microscope.

- 1. **Mouse Safety Evaluation**

BALB/c mice (6-8 weeks old, 18-22 g) will be used for the safety evaluation. At the end of the experiment, blood samples will be collected via retro-orbital bleeding or cardiac puncture under anesthesia. Biochemical Analysis: Serum will be separated and analyzed for liver function markers (ALT, AST). Hematology: Complete blood count (CBC) will be performed to assess hematological parameters such as red blood cell (RBC) count, white blood cell (WBC) count, hemoglobin, and platelet count. After blood collection, mice will be euthanized, and major organs (e.g., liver, kidney, heart, spleen, lung, and tumor tissue) will be harvested and fixed in 4% paraformaldehyde. Fixed organs will be embedded in paraffin, sectioned, and stained with HE. Histological examination will be performed to assess tissue integrity, signs of inflammation, necrosis, fibrosis, and any other pathological changes.

- 1. **Statistical Analysis**

All experimental data were analyzed using GraphPad Prism (version 9.0). Results are presented as mean ± SD. The statistical significance between two groups was determined using a two-tailed unpaired Student’s t-test. For comparisons involving more than two groups, one-way analysis of variance (ANOVA) followed by Tukey’s test was employed to identify significant differences. For survival analysis, Kaplan-Meier survival curves were generated, and the log-rank test was used to compare survival rates between groups. All p values are two-sided, and statistical significance was evaluated at the level of 0.05 (****p* < 0.001; ***p* < 0.01; **p* < 0.05).

**Supplementary Figures and Tables**

**Table S1**. HPLC analyses drug efficiency; Loading ratio and encapsulation efficiency of Dalpiciclib and Fluzoparib in a representative experiment.

|  | **Loading Efficiency** | **Encapsulation Efficiency** |
| --- | --- | --- |
| **Dalpiciclib** | **21.50%** | **75.23%** |
| **Fluzoparib** | **22.57%** | **79.01%** |


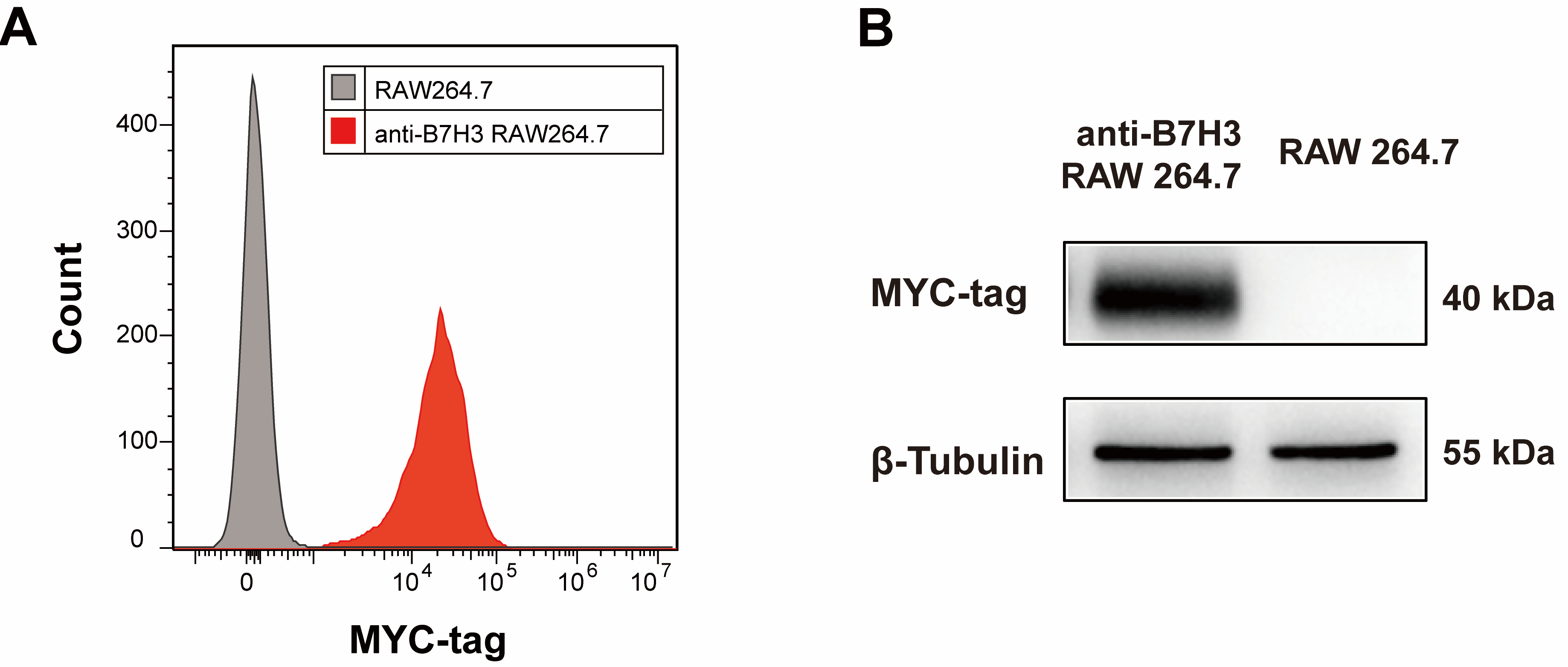


**Figure S1.** Validation of anti-B7H3 expression in RAW264.7. A) Flow cytometry of MYC-tag on the surface of transduced RAW264.7 cells. B) Western Blot of MYC-Tag in transduced RAW264.7 cells.


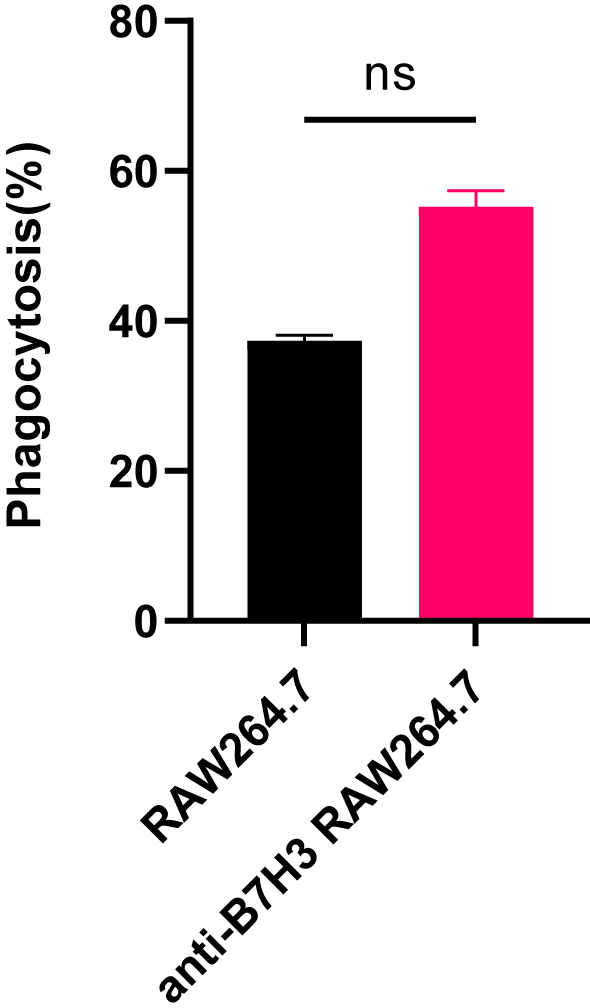


**Figure S2.** Quantification of phagocytosis percentage. The statistical analysis was performed with ANOVA analysis, **p* <0.05, ***p* <0.01, ****p* <0.001.


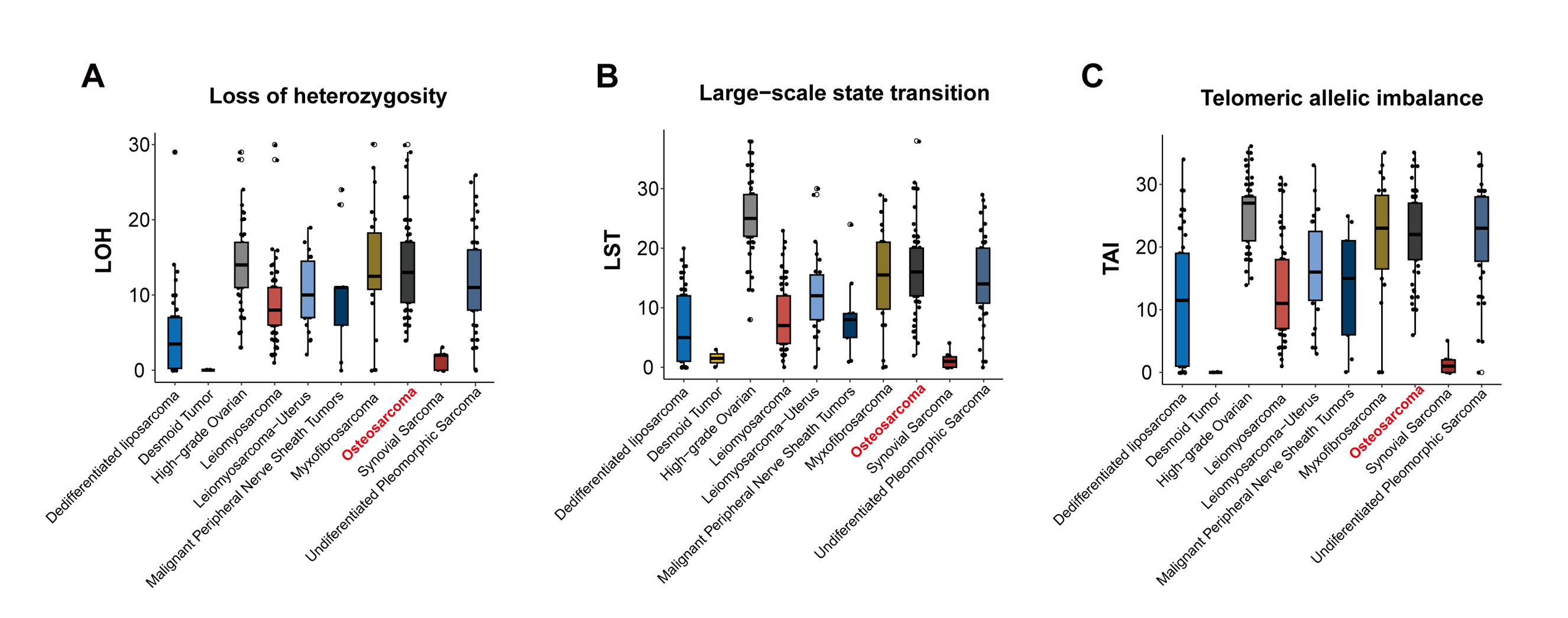


**Figure S3.** Analysis of genomic instability features in the TCGA and TARGET-OS cohorts (n = 377).


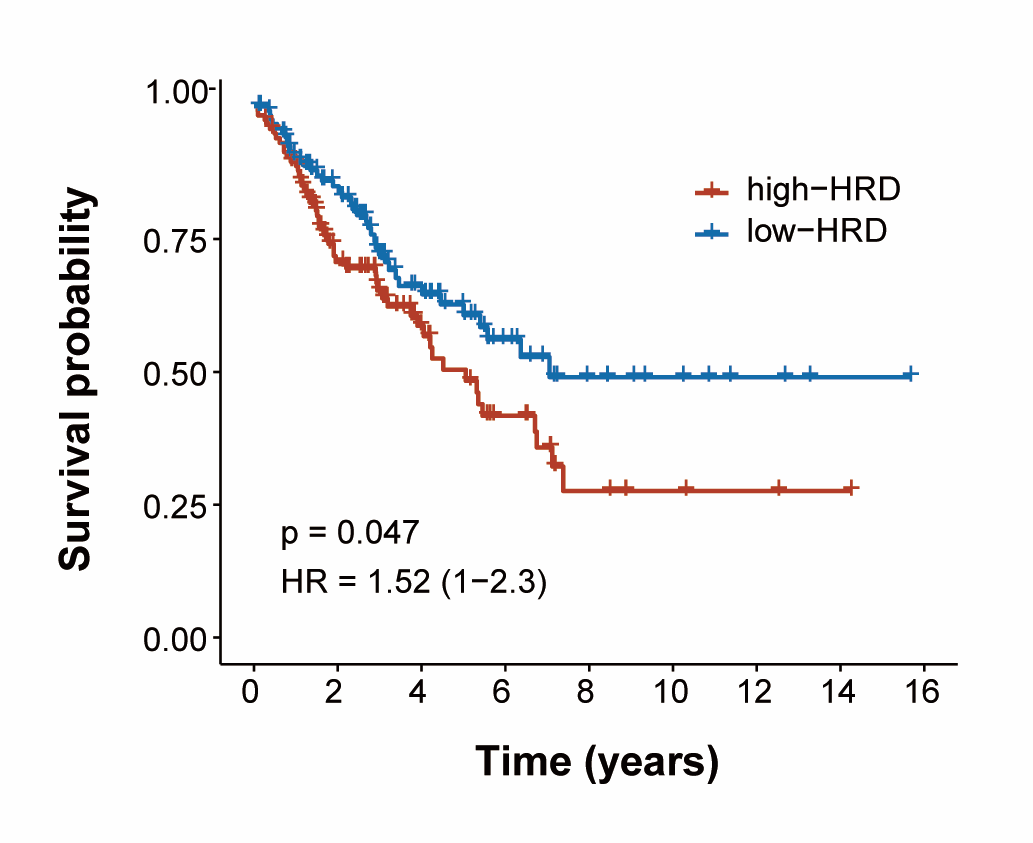


**Figure S4.** Survival analysis of HRD score in the TCGA-SARC cohort (n = 240).


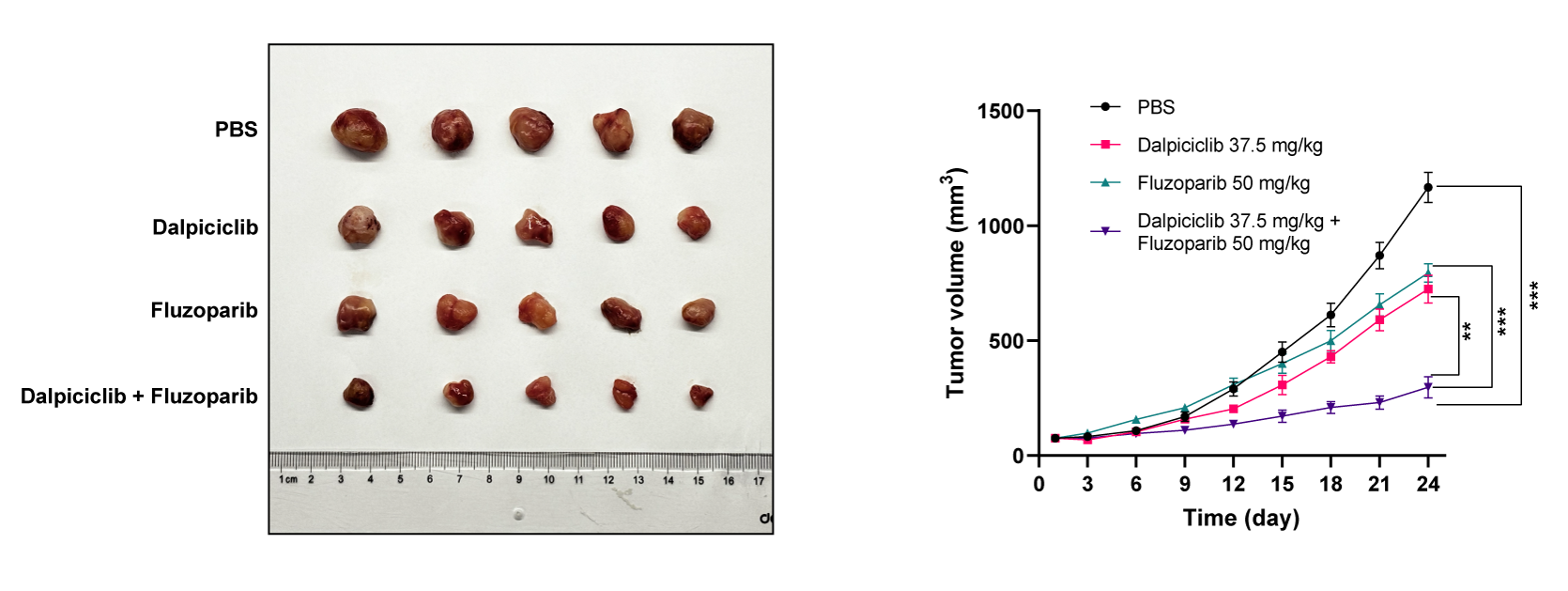


**Figure S5.** Photographs of tumor tissues at 24th day and Tumor Volume Curves. BALB/c mice (K7M2 cells) treated with Dalpicilib (37.5 mg/kg) and Fluzoarib (50 mg/kg), either alone or in combination (21 consecutive daily oral gavage; n = 5). Data are all presented as the mean ± SEM. The statistical analysis was performed with ANOVA analysis, **p* <0.05, ***p* <0.01, ****p* <0.001.


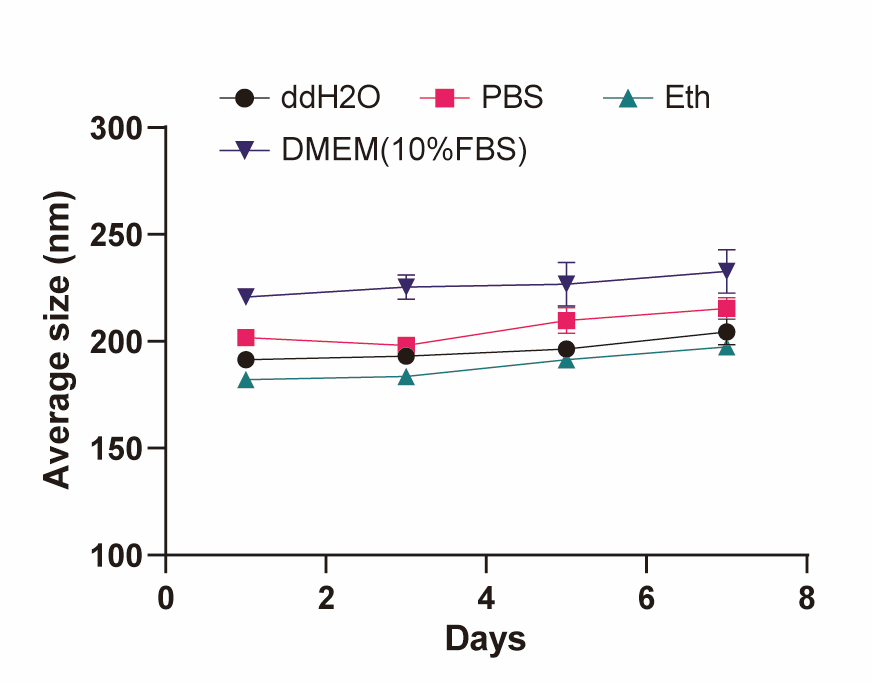


**Figure S6.** Particle size of the nanoparticles over seven days in various solvent systems (n = 3). Data are all presented as the mean ± SD.


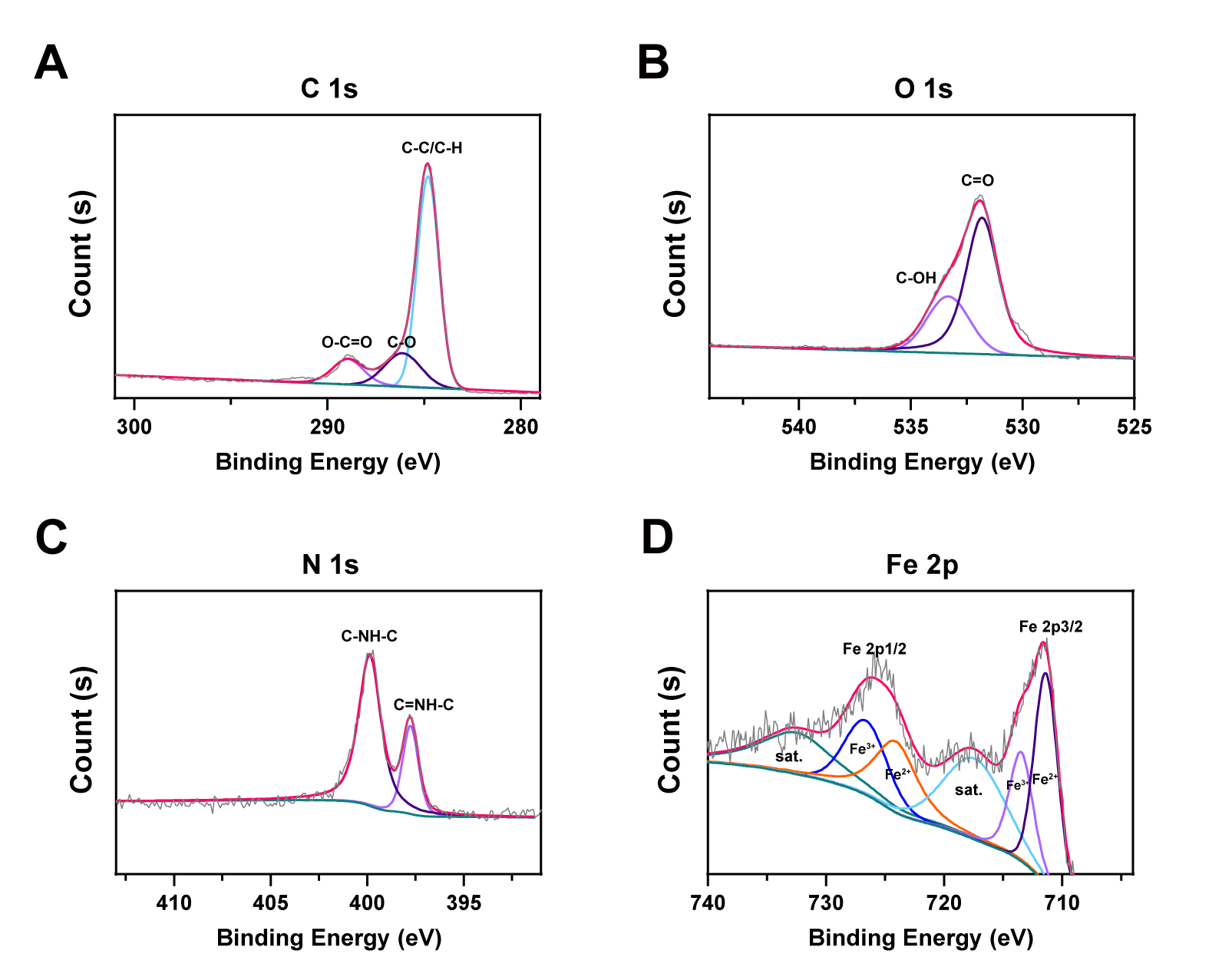


**Figure S7.** C 1s, O 1s, N 1s, and Fe 2p XPS spectra of MOFs.


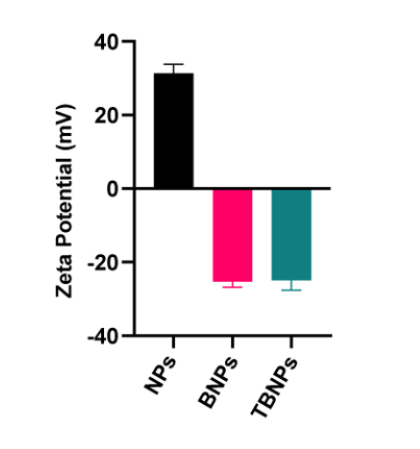


**Figure S8.** ZETA potential measurements for NPs, BNPs, and TBNPs (n = 3). Data are all presented as the mean ± SD.


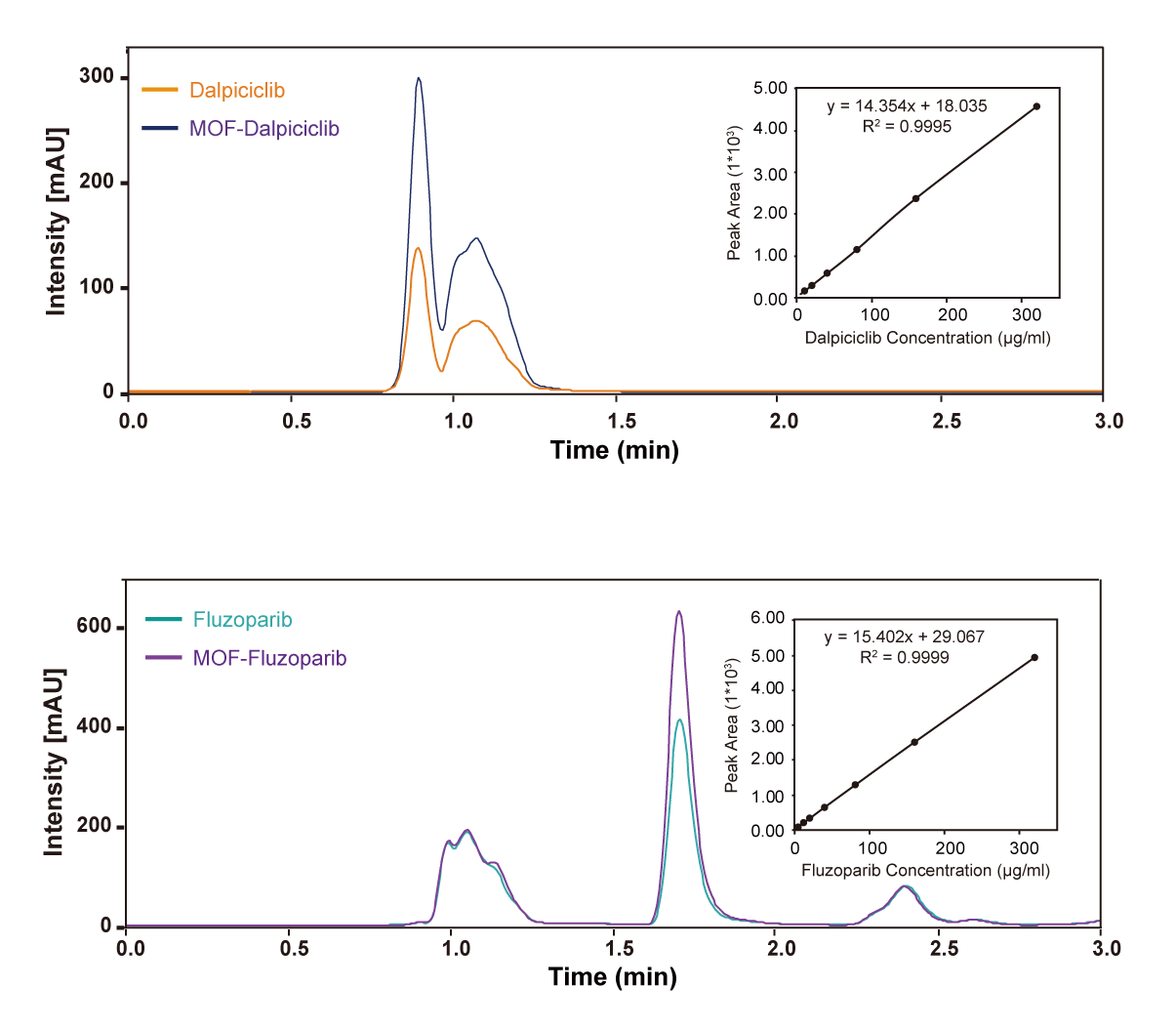


**Figure S9.** HPLC analysis for Dalpiciclib-loaded MOFs and Fluzoparib-loaded MOFs.


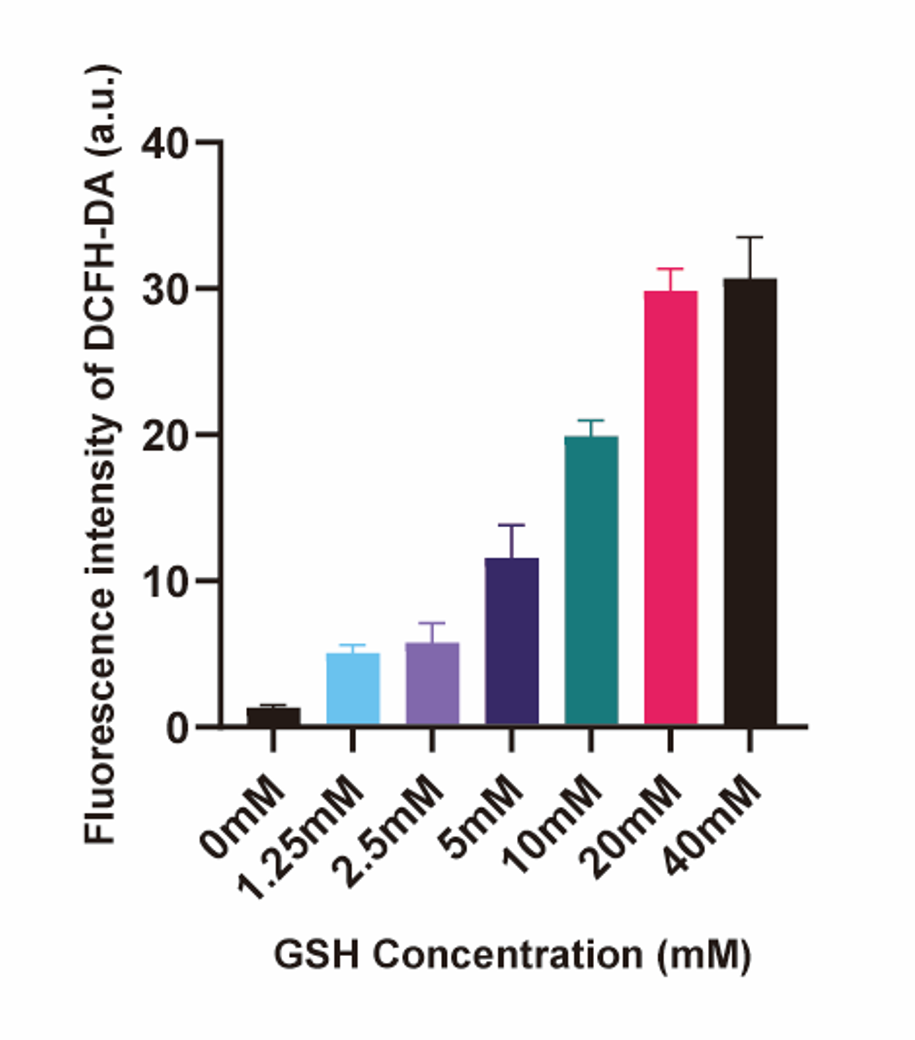


**Figure S10.** ROS production by NPs under irradiation at different GSH concentrations (n = 6). Data are all presented as the mean ± SD.


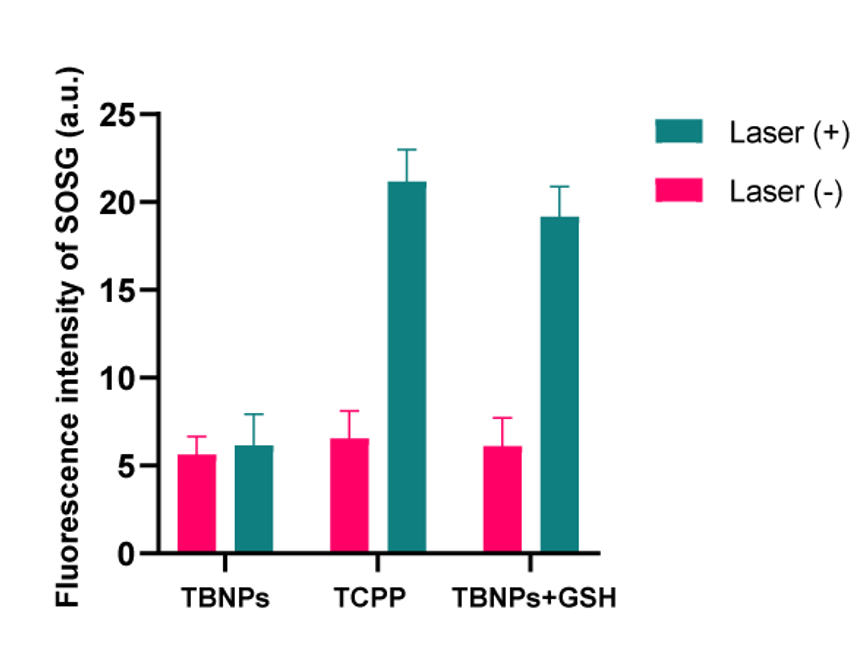


**Figure S11.** Fluorescence intensity of SOSG probes in different Groups (n = 6). Data are all presented as the mean ± SD.


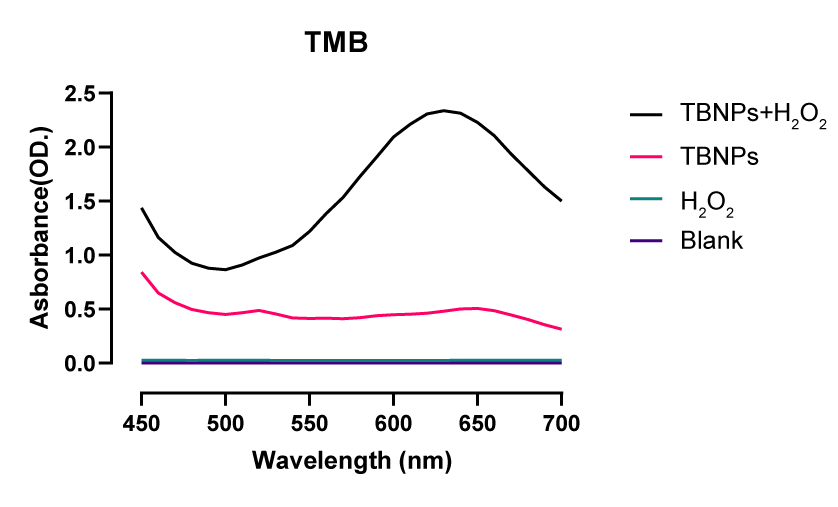


**Figure S12.** UV–vis absorption spectra of TMB in different groups (n = 6).


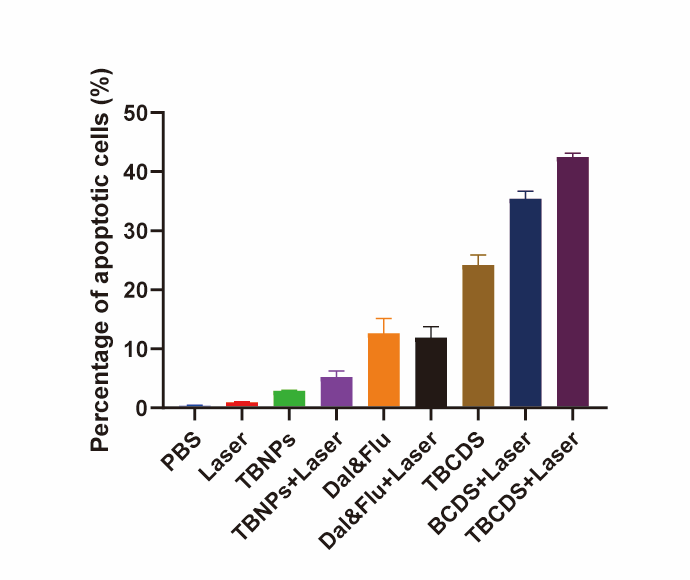


**Figure S13.** Quantification of apoptotic cells (Double positive for Annexin V and DAPI) in various treatment groups (n = 3). Apoptotic percentage of TBCDS **+** Laser vs. other groups: all *p* < 0.001**.** Data are all presented as the mean ± SD. The statistical analysis was performed with ANOVA analysis.


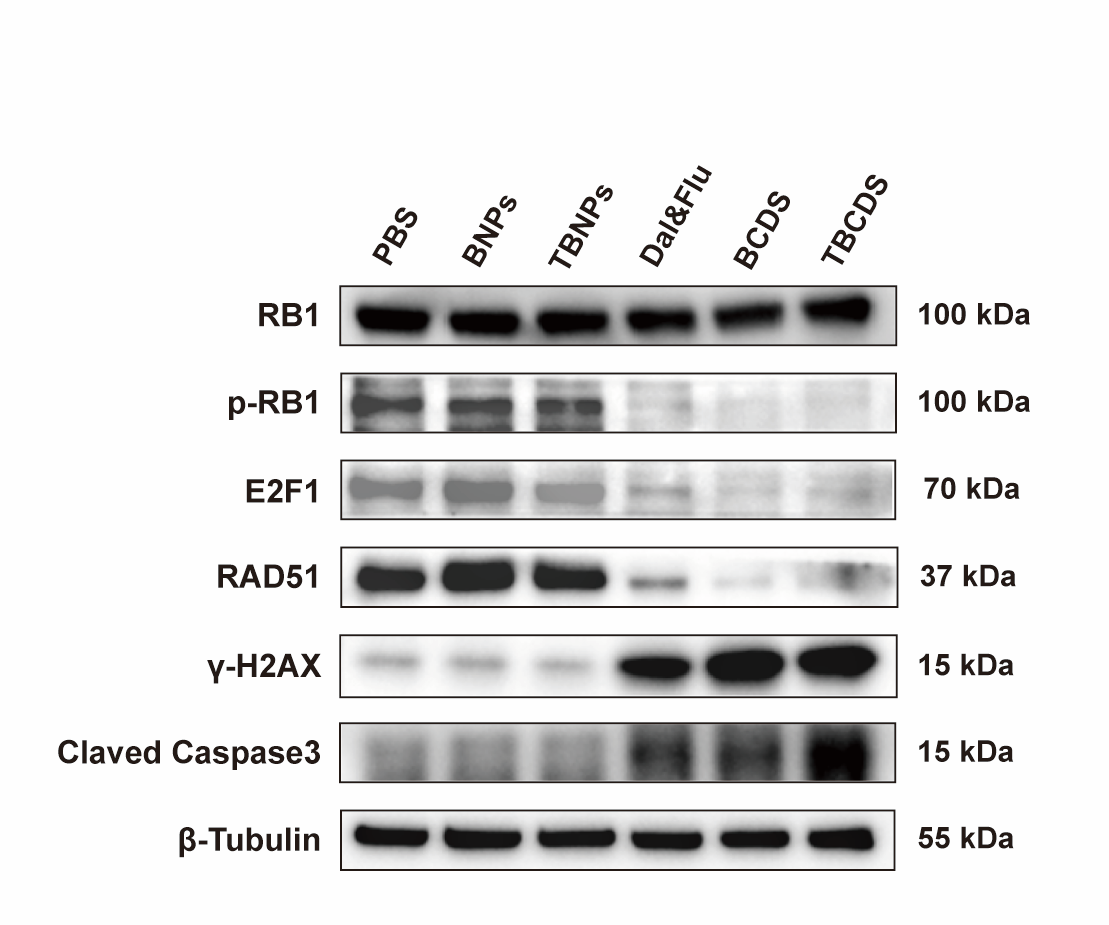


**Figure S14.** Representative western blot analysis of p-RB1, E2F1, RAD51, γ-H2AX, and cleaved Caspase 3 in SJSA1 after different treatments, n=3.


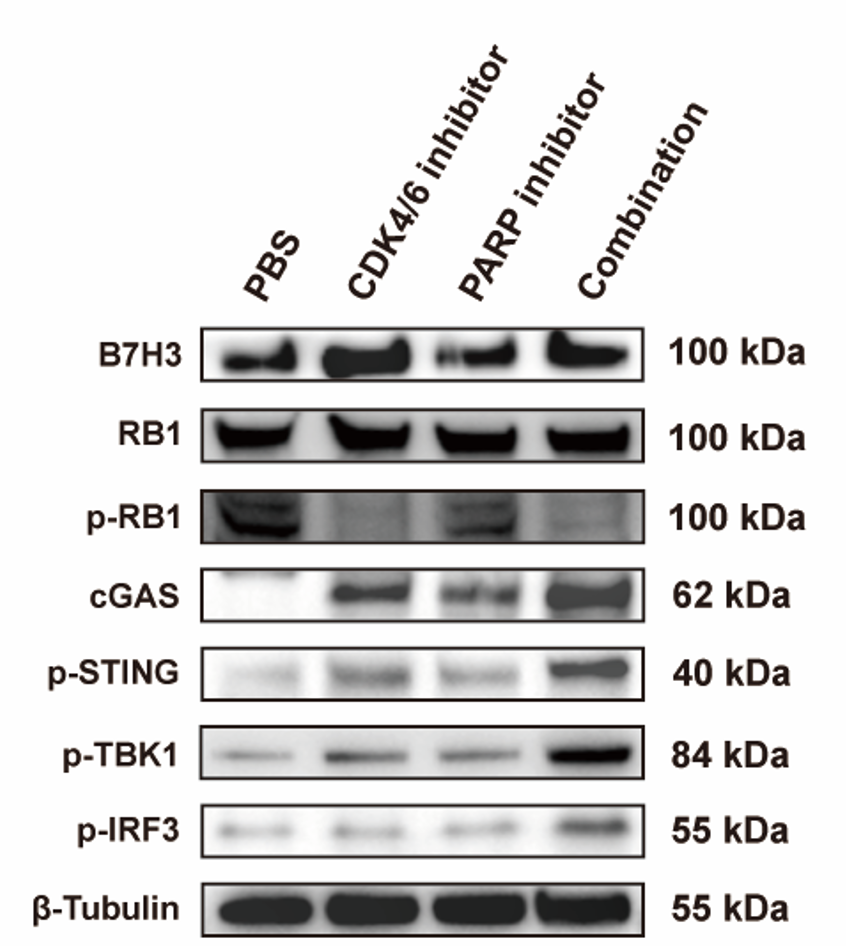


**Figure S15.** Representative western blot images of B7H3 and cGAS-STING proteins expression in SJSA1 cells treated with PBS, CDK4/6 inhibitor, PARP inhibitor, and combination therapy, n=3.


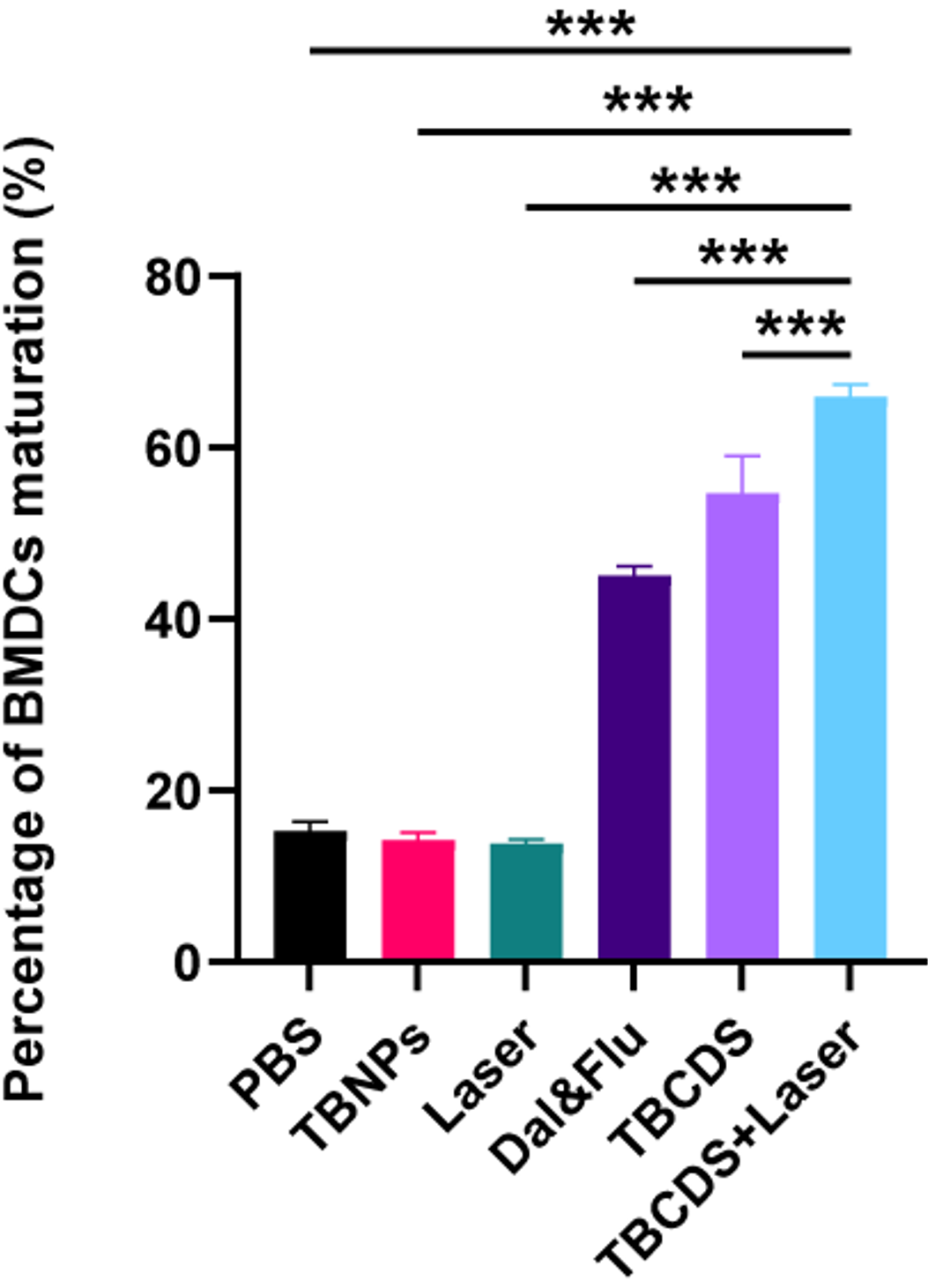


**Figure S16.** Quantitation analysis of BMDCs maturation in different groups. Data are all presented as the mean ± SD. The statistical analysis was performed with ANOVA analysis, **p* <0.05, ***p* <0.01, ****p* <0.001.


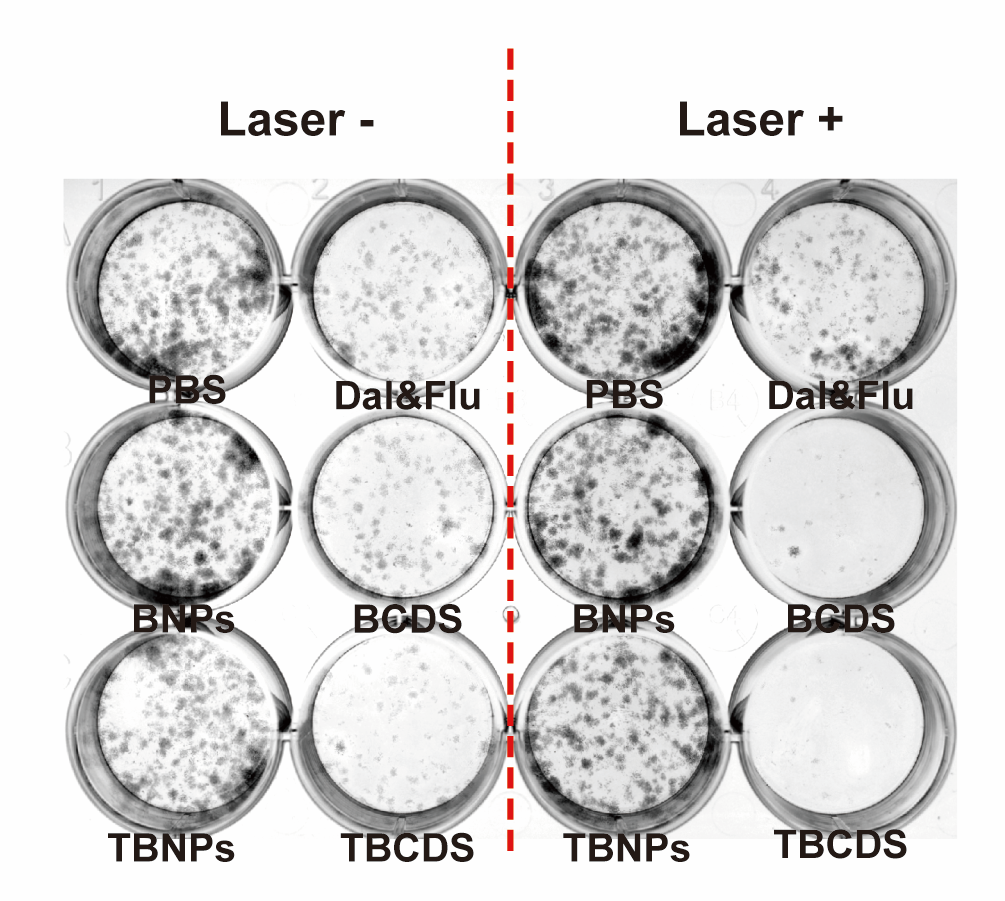


**Figure S17.** Clonogenic assay for SJSA1 in different treatment groups.


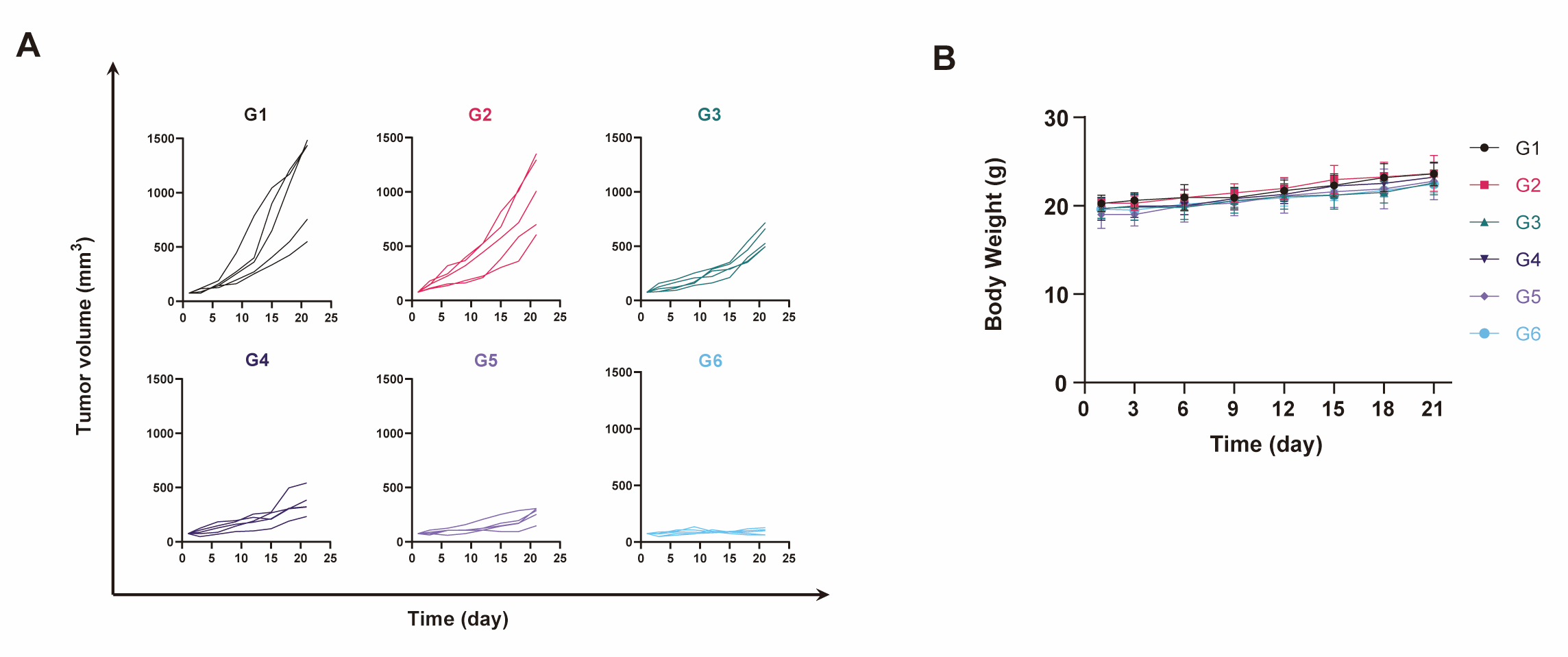


**Figure S18.** Individual Tumor volume growth curves and body weight changes in SJSA1 xenograft tumor-bearing NSG mice (n = 5). Data are all presented as the mean ± SD. The statistical analysis was performed with ANOVA analysis, **p* <0.05, ***p* <0.01, ****p* <0.001.


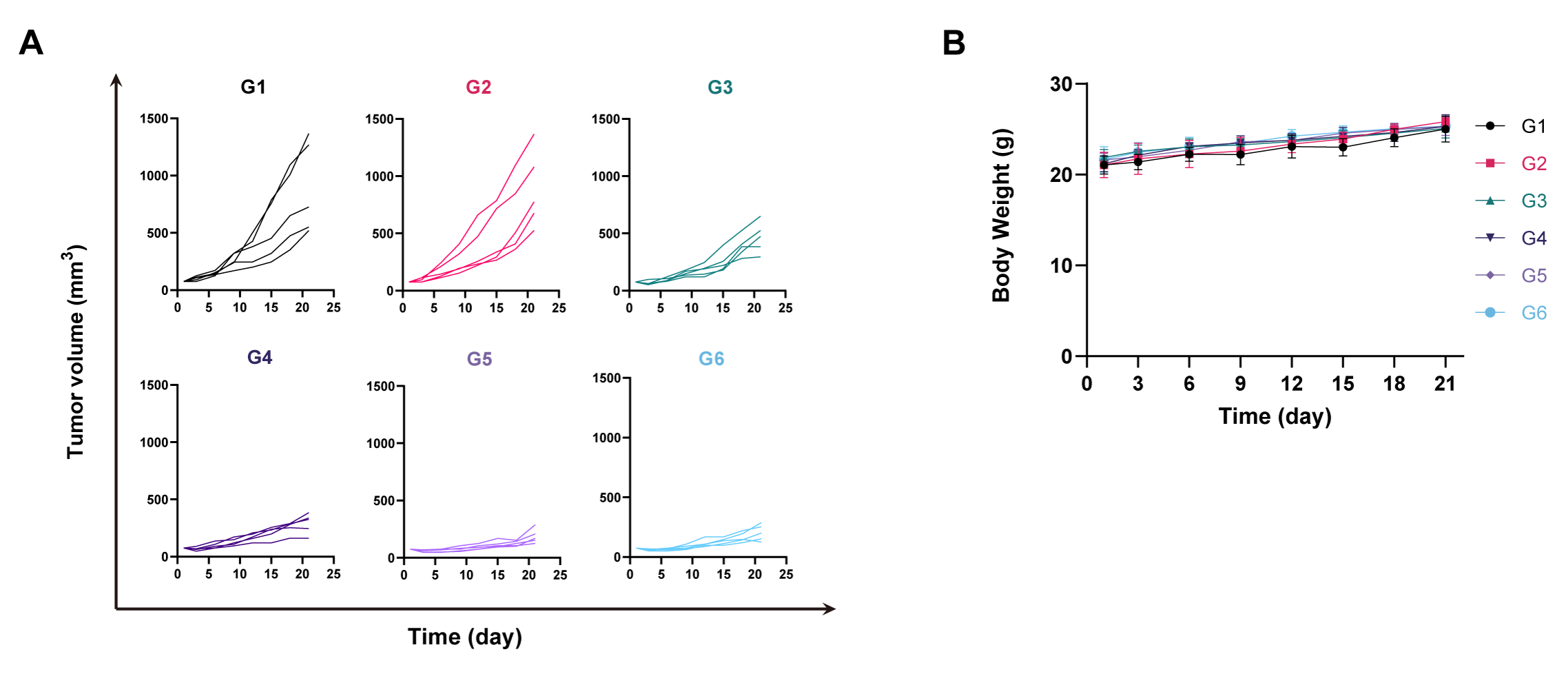


**Figure S19.** Individual Tumor volume growth curves and body weight changes in SJSA1-B7H3 knockdown xenograft tumor-bearing NSG mice (n = 5). Data are all presented as the mean ± SD. The statistical analysis was performed with ANOVA analysis, **p* <0.05, ***p* <0.01, ****p* <0.001.


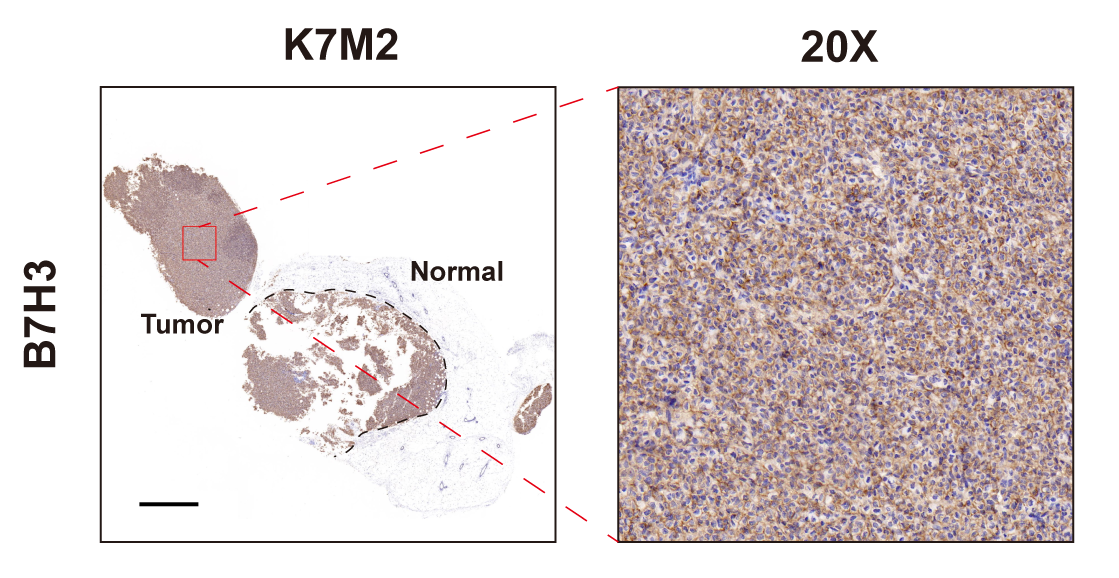


**Figure S20.** Immunohistochemical staining of B7H3 in K7M2 tumors (Scale bar = 1000 μm)**.**


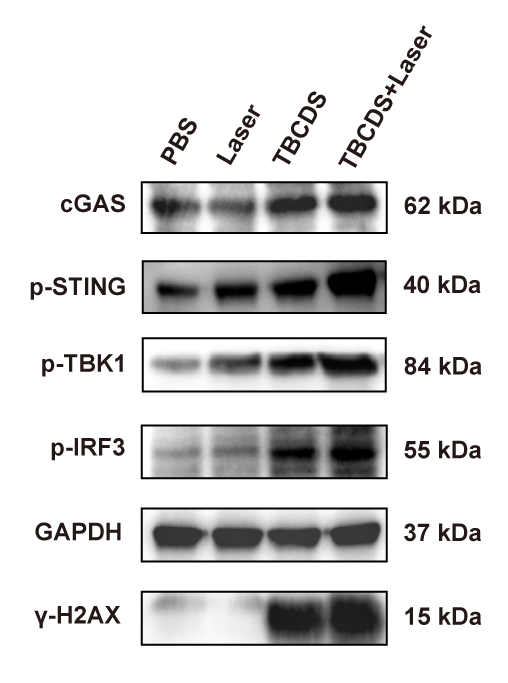


**Figure S21.** Representative western blot images of cGAS-STING proteins expression in mouse tumor treated different treatment groups, n = 3.


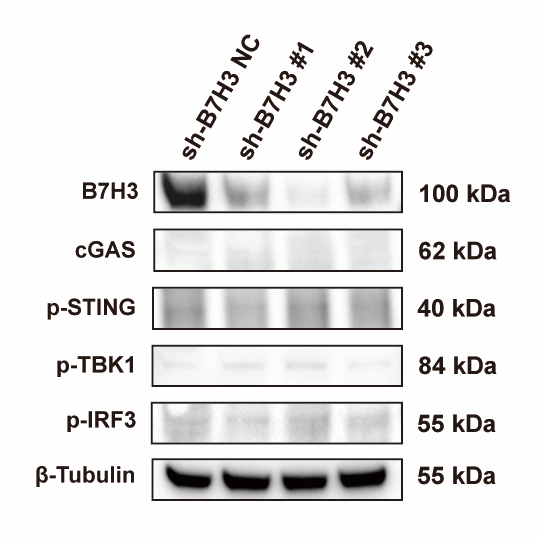


**Figure S22.** Representative western blot images of cGAS-STING proteins expression in B7H3-knockdown SJSA1 cells, n = 3.


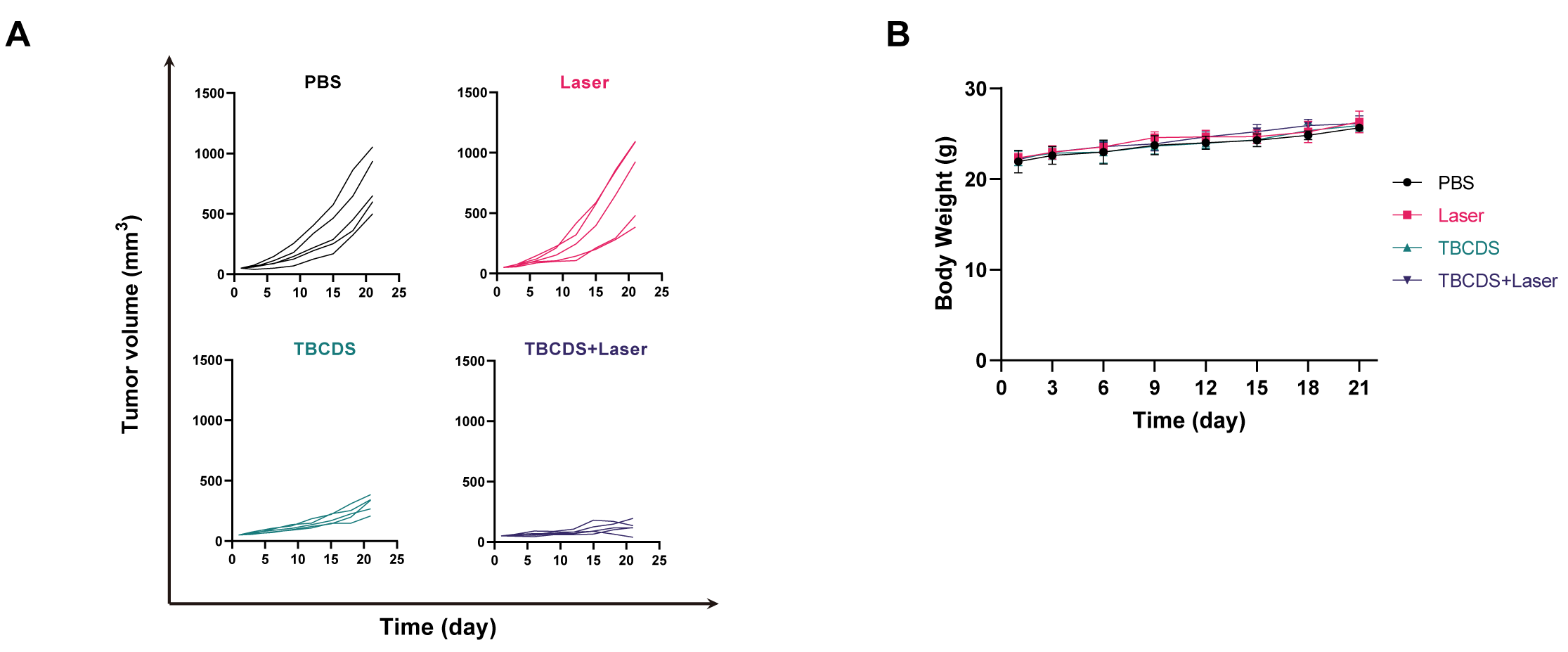


**Figure S23.** Individual Tumor volume growth curves and body weight changes in PDX mice of osteosarcoma (n = 5). Data are all presented as the mean ± SD. The statistical analysis was performed with ANOVA analysis, **p* <0.05, ***p* <0.01, ****p* <0.001.


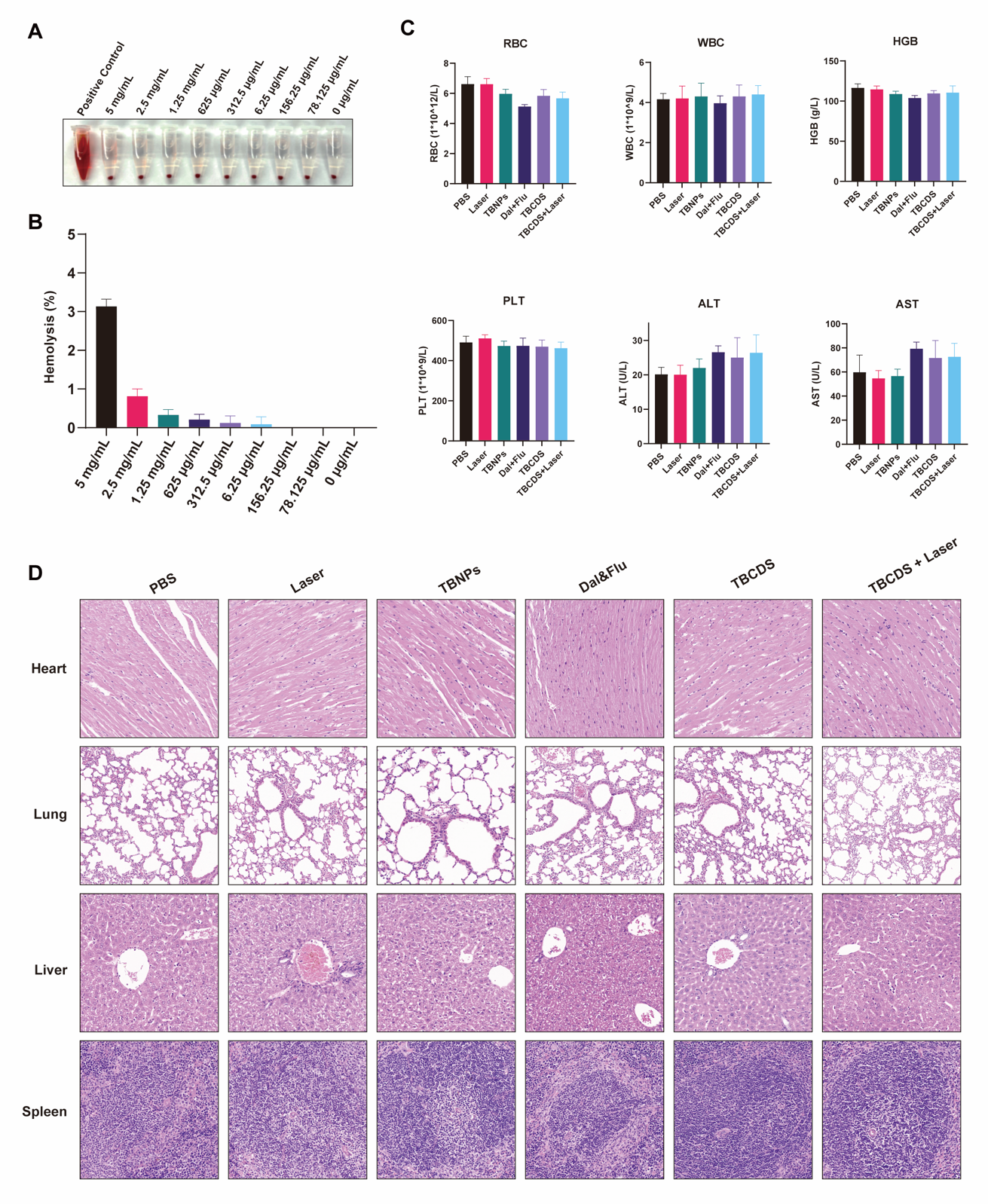


**Figure S24**. **Safety Assessment of Nanoparticles in BALB/c Mice.** A) Hemolysis assay testing various concentrations of nanoparticles to assess hemolytic activity on mouse blood samples. B) Quantitative analysis of hemolysis percentage for different nanoparticle concentrations groups (n = 6). C) Complete blood count (CBC) and liver enzyme analysis, presenting Red Blood Cell count (RBC), White Blood Cell count (WBC), Hemoglobin (HGB), Platelet count (PLT), Alanine Aminotransferase (ALT), and Aspartate Aminotransferase (AST) levels in mice from different treatment groups (n = 5): PBS, Laser, TBNPs, Dal&Flu, TBCDS, and TBCDS + Laser. D) Histological examination of major organs, including heart, lung, liver, and spleen, from treated mice. Organs were stained with Hematoxylin and Eosin (H&E) to assess potential tissue damage or abnormalities. Data are all presented as the mean ± SD. The statistical analysis was performed with ANOVA analysis, **p* <0.05, ***p* <0.01, ****p* <0.001.
